# Supplementary material for: wg-blimp: an end-to-end analysis pipeline for whole genome bisulfite sequencing data
Source: BMC Bioinformatics. 2020 May 1;21:169. doi: 10.1186/s12859-020-3470-5 (PMC7195798; doi:10.1186/s12859-020-3470-5)
Supplement: Supplementary file 1 — Additional file 1 Supplementary Material [file 12859_2020_3470_MOESM1_ESM.pdf]

wg-blimp: an end-to-end analysis pipeline for whole genome bisulfite sequencing data
Supplementary Material

Marius Wöste, Elsa Leitão,
Sandra Laurentino, Bernhard Horsthemke,
Sven Rahmann and Christopher Schröder

January 30, 2020

1 Shiny interface

We implemented a web application to ease sharing our WGBS analysis results with multiple researchers across multiple institutes and to simplify access to BAM files for inspection using the IGV. We chose the R Shiny framework for our analysis as it provides necessary functionality conveniently through R, such as loading and handling tables using the `data.table` package and creating plots with `ggplot2`.

The wg-blimp user interface consists of five separate tabs for dataset selection, quality control statistics, pipeline parameters, overview over called DMRs and UMR/LMR/PMD segmentation.

1.1 Dataset selection

All WGBS datasets analysed by the wg-blimp analysis pipeline may be loaded into the Shiny user interface for sharing with other researchers. Figure S1 shows the corresponding part of the interface.

1.2 QC statistics

The wg-blimp workflow performs quality control checks using FastQC, Picard, Qualimap, MultiQC and gathers methylation reports from MethylDackel. The shiny interface provides a brief overview over the gathered metrics and links to more detailed MultiQC and Qualimap reports. Links to alignment data for use with the IGV are also provided here. Figure S2 shows the statistics tab of the interface.

1.3 Pipeline parameters

Results generated by bioinformatics pipelines commonly depend on the parameters in use. We provide the config file along with the DMR analysis results to enable a more compre-

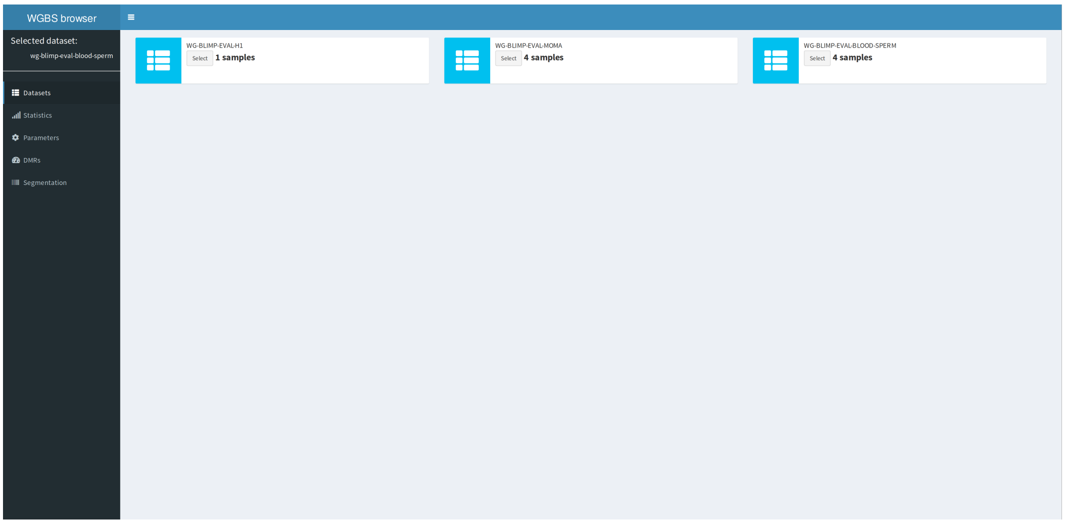

Figure S1: Shiny interface dataset selection tab.

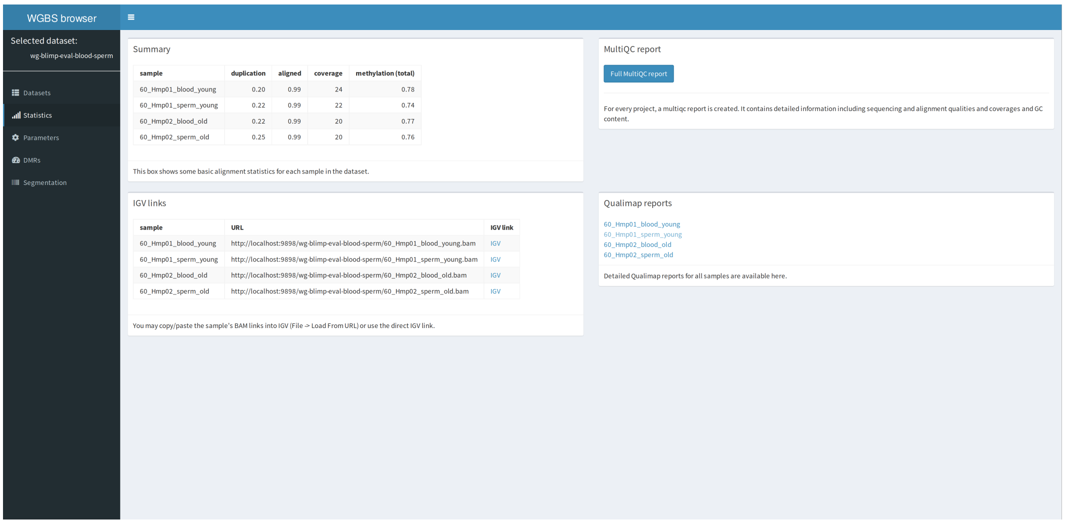

Figure S2: Shiny statistics tab.

hensive discussion of results. Figure S3 depicts the parameter overview tab.

1.4 DMRs

A tab to inspect DMRs retrieved by the calling pipeline is also provided. DMRs may be filtered by:

- Number of CpGs within DMR
- Absolute difference in methylation
- Length of DMR in bp

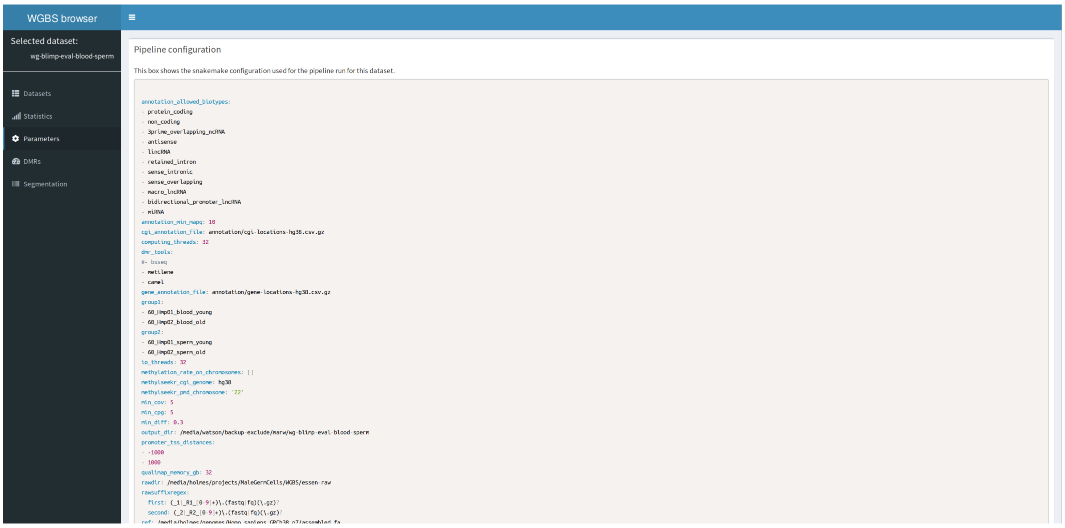

Figure S3: Shiny parameters tab.

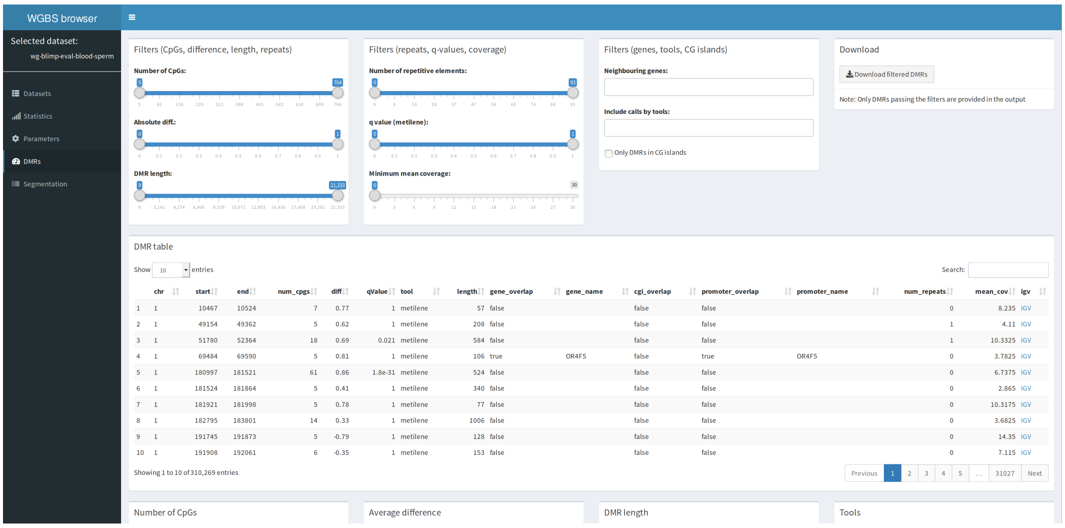

Figure S4: Shiny DMR tab.

- Number of repetitive elements within DMR
- q-value as reported by metilene
- Minimum mean coverage in DMR
- Genes (or promoters of genes) overlapped by DMRs
- Tools used for DMR calling
- Intersection with CG islands

Figure S4 gives an overview over the DMR tab. Filters are applied immediately and updated reactively through Shiny.

1.5 Segmentation

wg-blimp's MethylSeekR integration preemptively computes UMRs/LMRs with and without consideration of PMDs. α distributions are displayed to simplify decision whether the methylome at hand contains PMDs. If the distribution appears to be bimodal, one may assume PMDs to be present. The Shiny interface also provides information on the relation of number of CpGs to median methylation in each segment, as depicted in Figure 2. Usually UMRs are longer than LMRs, which should be distinguishable from the UMR/LMR heatmap. A plot displaying information on FDR and methylation cutoffs is also provided for quality control.

2 Analysis of public WGBS datasets using wg-blimp

We first applied wg-blimp to a published dataset of two isogenic pairs of human monocyte and macrophage samples. Details are described in the original publication (EGA accession EGAS00001001595) [1]. We also used wg-blimp to analyse a blood and sperm dataset to find differences in methylation between sperm and somatic cells. The dataset is also publicly available (ENA accession PRJEB28044). For this dataset pooled DNA from sperm and blood DNA (n=6 for each) was extracted from healthy young (age 18-24) and old (age 61-71) men, yielding four WGBS samples [2]. For both datasets 1% Lambda phage DNA was added for later quality control. Sequencing was performed on Illumina HiSeq 2500. Protocols for the H1 ESC sample are described in the original publication (NCBI SRA accession SRR3274347) [3].

2.1 Quality control

Table S1 shows the QC parameters as displayed in wg-blimp's shiny interface for each of the datasets.

Coverage profiles are $\geq 19\times$ and the mean methylation for lambda phage indicates successful bisulfite conversion for the monocyte/macrophage and blood/sperm datasets. Please note that lambda phage conversion rates are lacking for the H1 ESC sample as reads assigned to the lambda genome were discarded during upload to read repositories and thus not included in our computation. Since no QC parameter suggests errors, all samples were used for further analysis.

2.2 DMRs

The DMR algorithms used are expected to yield low numbers of calls for the monocyte/-macrophage dataset and high numbers for the blood/sperm comparison. Filtering may be performed in a trivial fashion through wg-blimp's user interface.

dataset	sample	duplication	aligned	coverage	conversion	methylation
monocyte / macrophage	43_Hm03_BlMa	0.23	1.00	22	0.98	0.80
monocyte / macrophage	43_Hm03_BlMo	0.19	1.00	21	0.99	0.80
monocyte / macrophage	43_Hm05_BlMa	0.23	1.00	33	0.99	0.81
monocyte / macrophage	43_Hm05_BlMo	0.22	1.00	33	0.99	0.81
blood / sperm	blood (young)	0.20	0.99	24	0.99	0.78
blood / sperm	sperm (young)	0.22	0.99	22	0.99	0.74
blood / sperm	blood (old)	0.22	0.99	20	0.99	0.77
blood / sperm	sperm (old)	0.25	0.99	20	1.00	0.76
H1 ESC	SRR3274347	0.05	1.00	19	-	0.76

Table S1: Quality control summary of analysed public datasets

It is notable that the number of DMRs called by wg-blimp is much higher for the monocyte/macrophage analysis in comparison to the original analysis (6 189 vs. 114 DMRs). We used three DMR calling algorithms while in the original study the authors utilized BSmooth for DMR calling which, in its default configuration, generates few, precise DMR calls. For our analysis we set lenient thresholds for the DMR calling to determine if we can still observe the expected global DMR properties such as CGI intersection and hypo-/hypermethylation. Users can change these parameters to control the number of DMR calls while increasing stringency. For example, if only DMR calls by metilene with q-value < 0.05 are accepted, only 15 DMRs remain for our DMR calling (5 of which overlap the 114 original calls). Similarly, bsseq only accounts for 23 DMR calls (15 of which overlap the original 114). camel produced 2522 calls, 112 of which overlap the original 114.

We also performed the monocyte/macrophage analysis using hg19 as reference to ensure the reference genome is not the primary cause of the difference in DMR calls. As expected, wg-blimp still yields a much higher number of calls. For hg19 a total of 6 691 DMRs is reported, with >90% of hg38 calls overlapping wg-blimp's lift-over hg19 calls. Of these 6 691 bsseq accounts for 29 DMRs, 18 of which overlap the original 114 calls. The remaining difference between our 29 bsseq DMRs and the original 114 BSmooth calls can be attributed to differences in alignment, methylation calling, bsseq configuration, and further development of the bsseq software itself.

Hence, the difference in the number of DMRs called can be explained by the configuration of DMR callers. bsseq performs strict calling, whereas metilene and camel produce more, albeit less precise DMRs. This comparison underlines the importance of tool and parameter choices for DMR calling, as it drastically impacts analysis results.

2.3 Segmentation

We focus on segmenting the H1 ESC methylome because of its previous analysis by the authors of the original MethylSeekR publication [4]. α distributions are similar for different chromosomes, so MethylSeekR infers the distribution for only a single chromosome. α smaller than 1 represents a polarized distribution favoring low and high methylation. α equal or greater than 1 suggests a uniform distribution. If an α distribution has a bimodal shape, we assume presence of PMDs. Since the α distribution appears to be unimodal

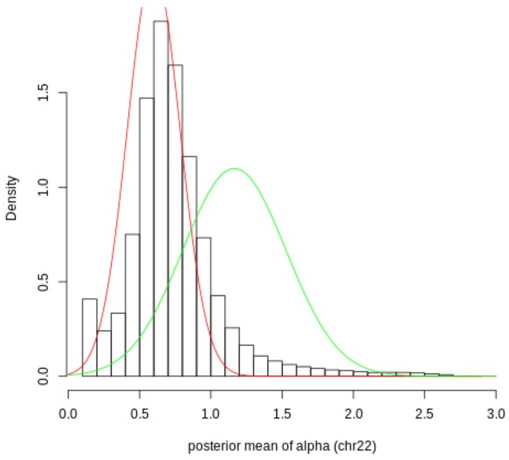

Figure S5: Distribution of α values for chromosome 22 of H1 ESC sample

with only few α values > 1, we do not consider PMDs for the H1 ESC sample, as depicted in Figure S5. This is consistent with the analysis of the original MethylSeekR authors where H1 cells showed a similar α distribution whereas, among other, fetal lung fibroblasts showed a clearly distinguishable bimodal α distribution [4]. Figure S6 shows intersection of LMRs/UMRs with exons and promoters. We here set promoters to the ranges around transcription start sites (TSSs): $[TSS - 1000, TSS + 1000]$. As expected, while 82.32% of UMRs are intersecting promoters, only 12.29% of LMRs show promoter intersection. This is also coherent with the original MethylSeekR findings [4].

2.4 Computational requirements

wg-blimp logs run times and memory requirements for each step. The resulting plots from the monocyte/macrophage and blood/sperm analyses are displayed in Figures S7 to S12.

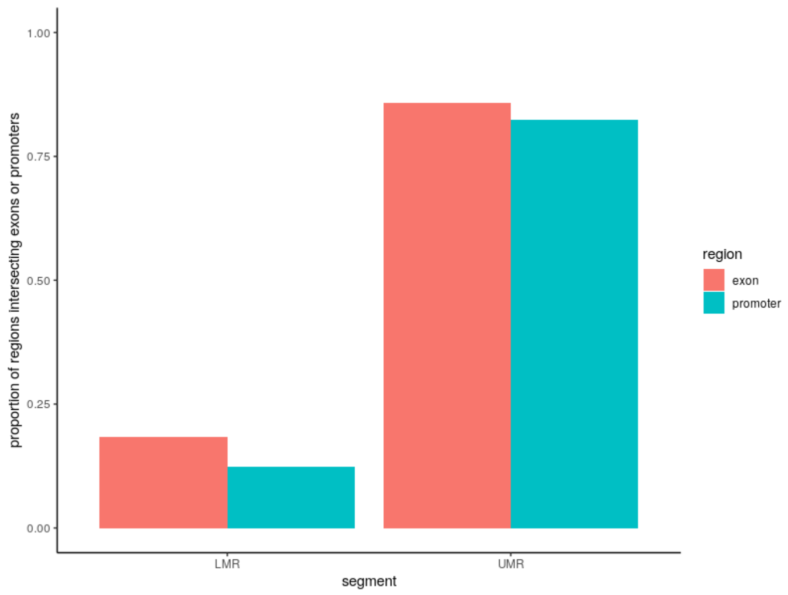

Figure S6: Intersection of LMRs/UMRs with exons and promoters

2.5 Parameters

This section shows the configuration files and environment used for the analyses.

2.5.1 Monocyte/Macrophage configuration file

```
annotation_allowed_biotypes:
- protein_coding
- non_coding
- 3prime_overlapping_ncRNA
- antisense
- lincRNA
- retained_intron
- sense_intronic
- sense_overlapping
- macro_lncRNA
- bidirectional_promoter_lncRNA
- miRNA
annotation_min_mapq: 10
bsseq_local_correct: false
cgi_annotation_file: annotation/cgi-locations-hg38.csv.gz
computing_threads: 16
dmr_tools:
- bsseq
- camel
- metilene
gene_annotation_file: annotation/gene-locations-hg38.csv.gz
group1:
- 43_Hm03_BlMa
- 43_Hm05_BlMa
group2:
- 43_Hm03_BlMo
- 43_Hm05_BlMo
io_threads: 16
methylation_rate_on_chromosomes: []
methylseekr_cgi_genome: hg38
methylseekr_fdr_cutoff: 5
methylseekr_methylation_cutoff: 0.5
methylseekr_pmd_chromosome: '22'
min_cov: 5
min_cpg: 4
min_diff: 0.3
output_dir: /media/watson/backup-exclude/marw/wg-blimp-eval-moma
promoter_tss_distances:
- -1000
- 1000
```

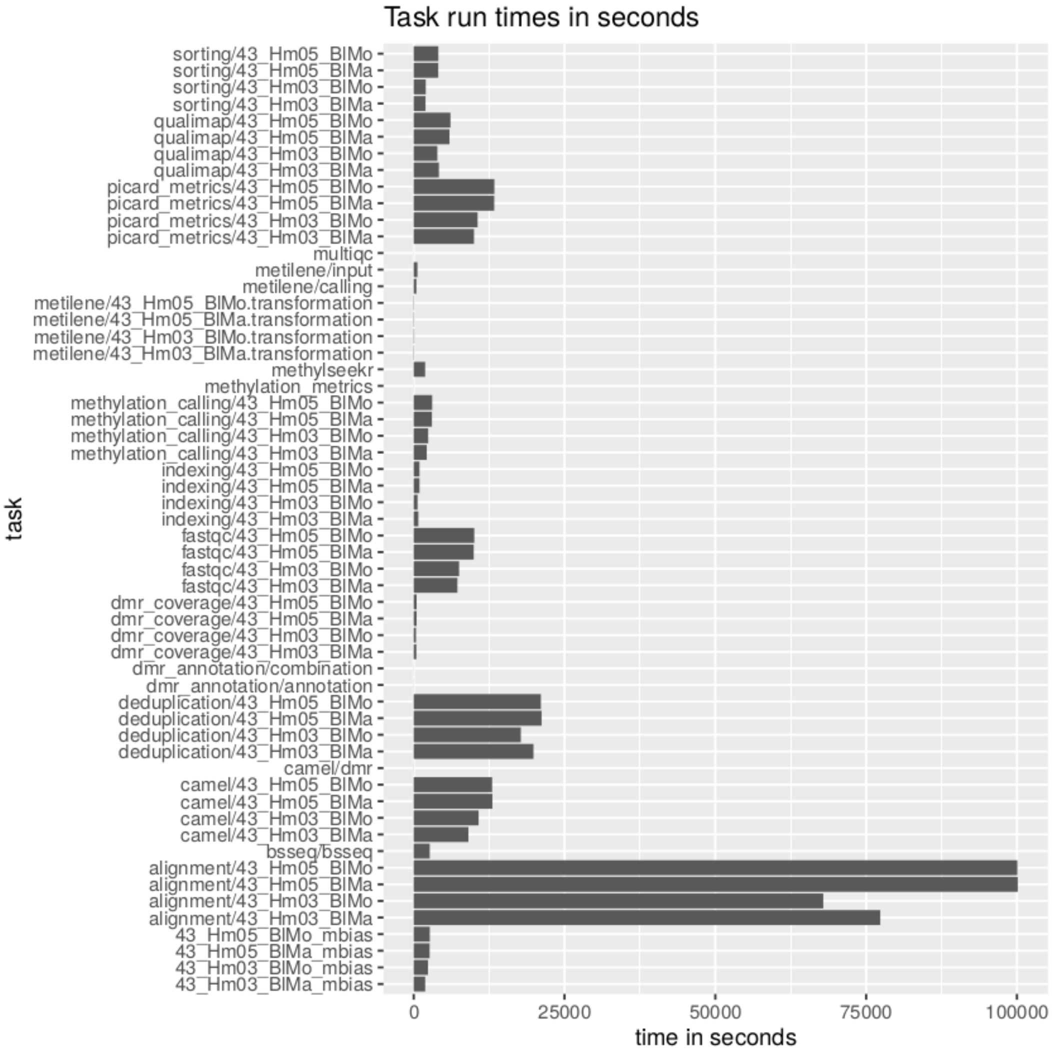

Figure S7: wg-blimp task run times for monocyte/macrophage dataset

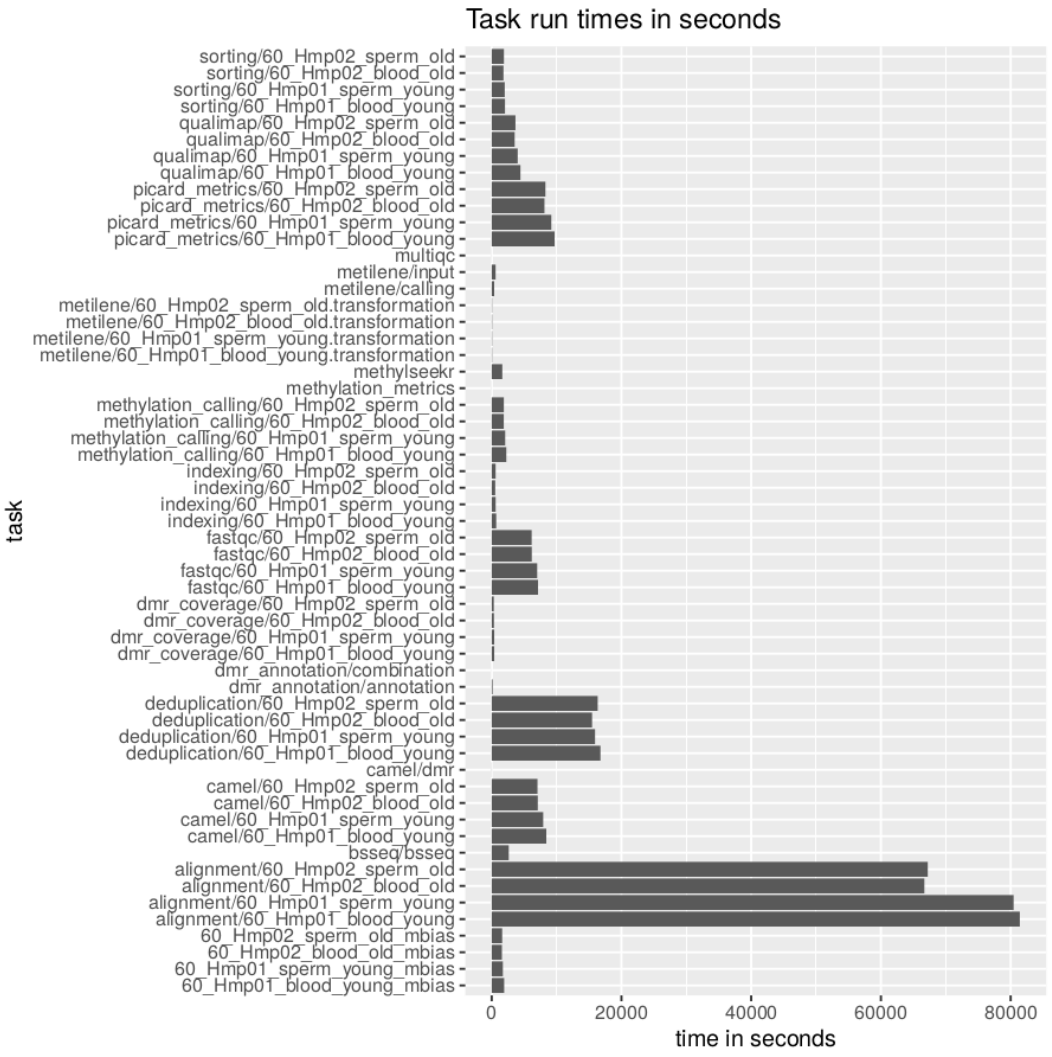

Figure S8: wg-blimp task run times for blood/sperm dataset

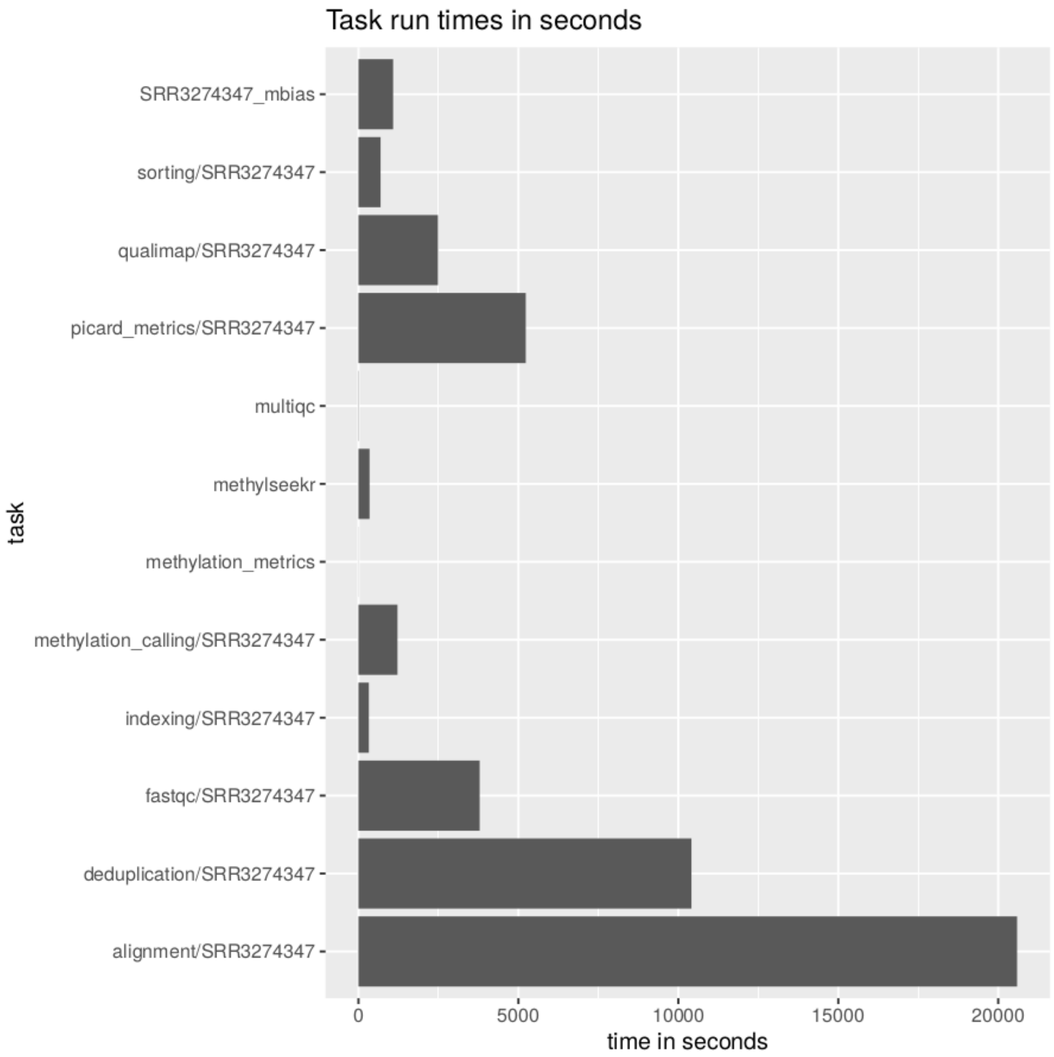

Figure S9: wg-blimp task run times for H1 ESC sample

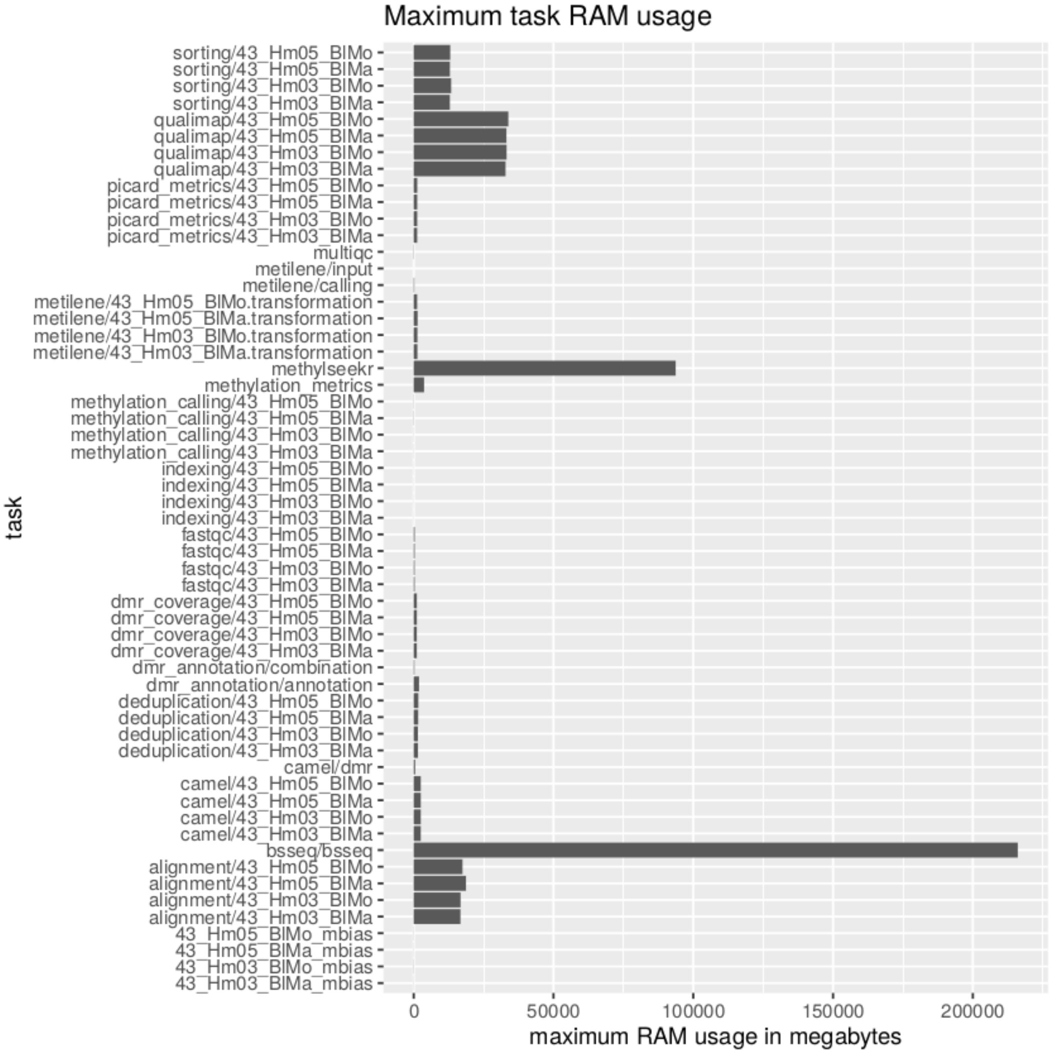

Figure S10: wg-blimp memory usages for monocyte/macrophage dataset

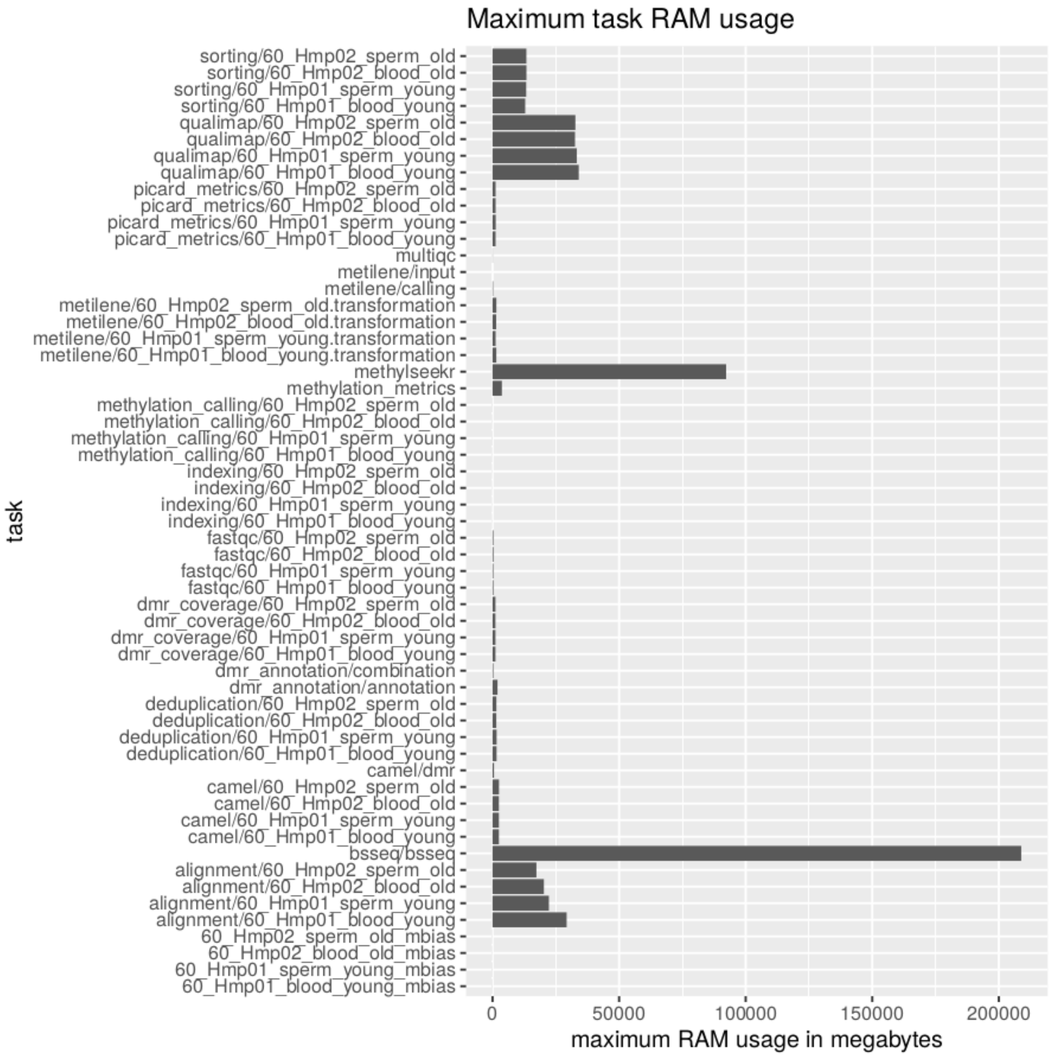

Figure S11: wg-blimp memory usages for blood/sperm dataset

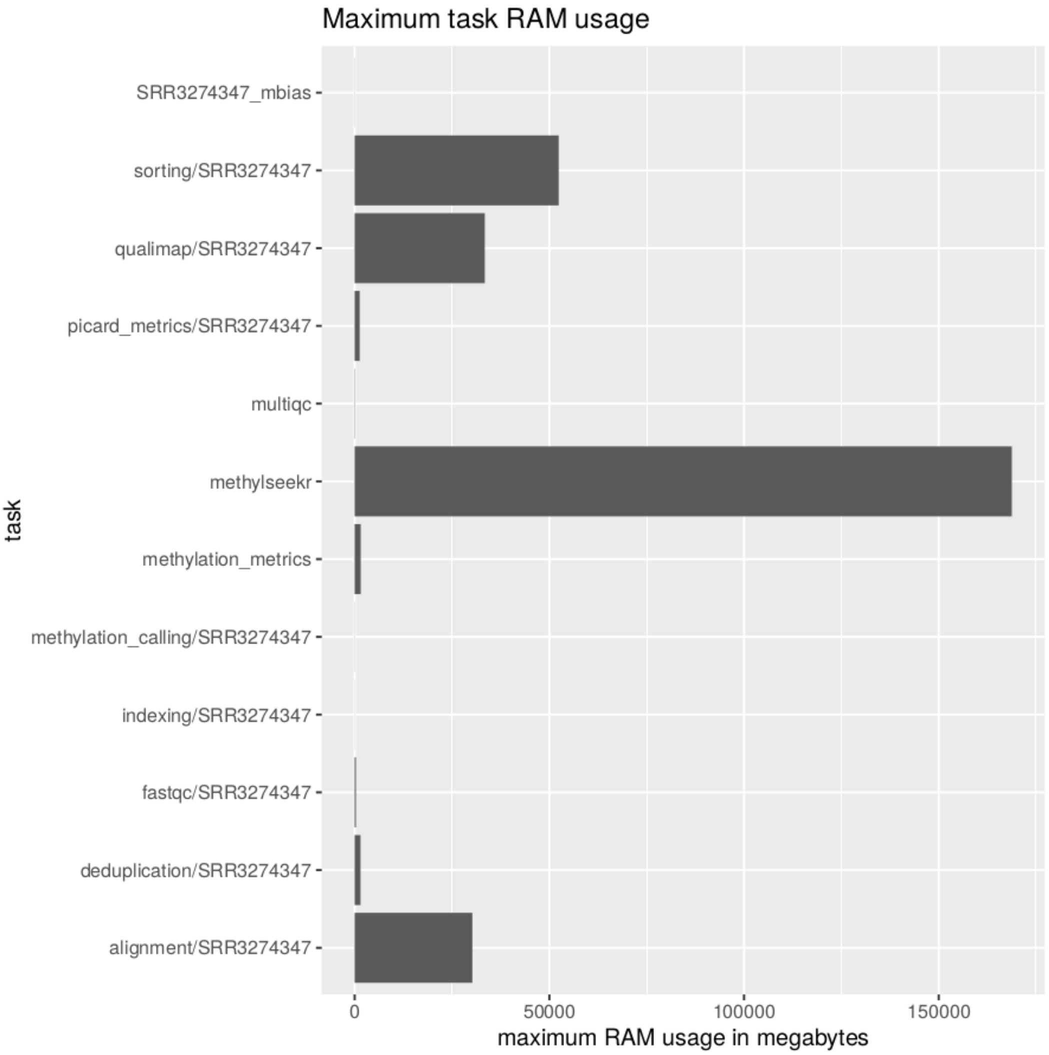

Figure S12: wg-blimp memory usages for H1 ESC sample

```
qualimap_memory_gb: 32
rawdir: /media/holmes/projects/MaleGermCells/WGBS/essen-raw
rawsuffixregex:
  first: (_1|_R1_[0-9]+)\.(fastq|fq)(\.gz)?
  second: (_2|_R2_[0-9]+)\.(fastq|fq)(\.gz)?
ref: /media/holmes/genomes/Homo_sapiens.GRCh38.p7/assembled.fa
repeat_masker_annotation_file: annotation/repeat-masker-hg38.csv.gz
repeat_masker_links:
  annotation/repeat-masker-hg19.csv.gz: https://uni-muenster.sciebo.de/s/tFPOT7weAc5eGLG/download
  annotation/repeat-masker-hg38.csv.gz: https://uni-muenster.sciebo.de/s/LwMik3kKY83oVT0/download
samples:
- 43_Hm03_BlMa
- 43_Hm05_BlMa
- 43_Hm03_BlMo
- 43_Hm05_BlMo
target_files:
- dmr/annotated-dmrs.csv
- qc/multiqc_report.html
- qc/methylation_metrics.csv
- segmentation/umr-lmr-all.csv
transcript_start_site_file: annotation/transcription-start-sites-hg38.csv.gz
```


2.5.2 Blood/Sperm configuration file


```
annotation_allowed_biotypes:
- protein_coding
- non_coding
- 3prime_overlapping_ncRNA
- antisense
- lincRNA
- retained_intron
- sense_intronic
- sense_overlapping
- macro_lncRNA
- bidirectional_promoter_lncRNA
- miRNA
annotation_min_mapq: 10
bsseq_local_correct: false
cgi_annotation_file: annotation/cgi-locations-hg38.csv.gz
computing_threads: 16
dmr_tools:
- bsseq
- camel
- metilene
gene_annotation_file: annotation/gene-locations-hg38.csv.gz
group1:
- 60_Hmp01_blood_young
- 60_Hmp02_blood_old
group2:
- 60_Hmp01_sperm_young
- 60_Hmp02_sperm_old
io_threads: 16
methylation_rate_on_chromosomes: ['LAMBDA']
methylseekr_cgi_genome: hg38
methylseekr_fdr_cutoff: 5
methylseekr_methylation_cutoff: 0.5
methylseekr_pmd_chromosome: '22'
min_cov: 5
min_cpg: 4
min_diff: 0.3
output_dir: /media/watson/backup-exclude/marw/wg-blimp-eval-blood-sperm
promoter_tss_distances:
- -1000
- 1000
qualimap_memory_gb: 32
rawdir: /media/holmes/projects/MaleGermCells/WGBS/essen-raw
rawsuffixregex:
  first: (_1|_R1_[0-9]+)\.(fastq|fq)(\.gz)?
  second: (_2|_R2_[0-9]+)\.(fastq|fq)(\.gz)?
ref: /media/holmes/genomes/Homo_sapiens.GRCh38.p7/assembled.fa
repeat_masker_annotation_file: annotation/repeat-masker-hg38.csv.gz
repeat_masker_links:
  annotation/repeat-masker-hg19.csv.gz: https://uni-muenster.sciebo.de/s/tFPOT7weAc5eGLG/download
  annotation/repeat-masker-hg38.csv.gz: https://uni-muenster.sciebo.de/s/LwMik3kKY83oVT0/download
samples:
- 60_Hmp01_blood_young
- 60_Hmp02_blood_old
- 60_Hmp01_sperm_young
- 60_Hmp02_sperm_old
target_files:
- dmr/annotated-dmrs.csv
- qc/multiqc_report.html
- qc/methylation_metrics.csv
- segmentation/umr-lmr-all.csv
```

```
transcript_start_site_file: annotation/transcription-start-sites-hg38.csv.gz
```

2.5.3 H1 ESC configuration file

```
annotation_allowed_biotypes:
- protein_coding
- non_coding
- 3prime_overlapping_ncRNA
- antisense
- lincRNA
- retained_intron
- sense_intronic
- sense_overlapping
- macro_lncRNA
- bidirectional_promoter_lncRNA
- miRNA
annotation_min_mapq: 10
bsseq_local_correct: false
cgi_annotation_file: annotation/cgi-locations-hg38.csv.gz
computing_threads: 64
dmr_tools:
- bsseq
gene_annotation_file: annotation/gene-locations-hg38.csv.gz
group1:
- SRR3274347
group2:
- SRR3274347
io_threads: 64
methylation_rate_on_chromosomes: []
methylseekr_cgi_genome: hg38
methylseekr_fdr_cutoff: 5
methylseekr_methylation_cutoff: 0.5
methylseekr_pmd_chromosome: '22'
min_cov: 5
min_cpg: 5
min_diff: 0.3
output_dir: /media/holmes/backup-exclude/wg-blimp-eval-h1
promoter_tss_distances:
- -1000
- 1000
qualimap_memory_gb: 32
rawdir: /media/holmes/backup-exclude/wg-blimp-eval-h1/fastq
rawsuffixregex:
  first: (_1|_R1_[0-9]+)\.(fastq|fq)(\.gz)?
  second: (_2|_R2_[0-9]+)\.(fastq|fq)(\.gz)?
ref: /media/holmes/genomes/Homo_sapiens.GRCh38.p7/assembled.fa
repeat_masker_annotation_file: annotation/repeat-masker-hg38.csv.gz
repeat_masker_links:
  annotation/repeat-masker-hg19.csv.gz: https://uni-muenster.sciebo.de/s/tFPOT7weAc5eGLG/download
  annotation/repeat-masker-hg38.csv.gz: https://uni-muenster.sciebo.de/s/LwMik3kKY83oVT0/download
samples:
- SRR3274347
target_files:
- qc/multiqc_report.html
- qc/methylation_metrics.csv
- segmentation/umr-lmr-all.csv
transcript_start_site_file: annotation/transcription-start-sites-hg38.csv.gz
```

2.5.4 wg-blimp conda environment

```
Name                          Version          Build    Channel
_libgcc_mutex                 0.1               main
_r-mutex                      1.0.1      anacondar_1    conda-forge
aioeasywebdav                 2.4.0       py36_1000     conda-forge
aiohttp                       3.5.4   py36h14c3975_0    conda-forge
alsa-lib                      1.1.5   h516909a_1001     conda-forge
appdirs                       1.4.3             py_1    conda-forge
asn1crypto                    0.24.0       py36_1003    conda-forge
async-timeout                 3.0.1          py_1000    conda-forge
attrs                         19.1.0            py_0    conda-forge
bcrypt                        3.1.6   py36h516909a_1    conda-forge
bedtools                      2.28.0      hdf88d34_0    bioconda
binutils_impl_linux-64        2.31.1      h6176602_1
binutils_linux-64             2.31.1      h6176602_8
bioconductor-annotate         1.62.0          r36_1     bioconda
bioconductor-annotationdbi 1.46.0          r36_1      bioconda
bioconductor-beachmat         2.0.0   r36he1b5a44_1     bioconda
bioconductor-biobase          2.44.0  r36h516909a_1     bioconda
bioconductor-biocgenerics     0.30.0          r36_1     bioconda
bioconductor-biocparallel     1.18.0  r36he1b5a44_1     bioconda
bioconductor-biostrings       2.52.0  r36h516909a_1     bioconda
bioconductor-bsgenome         1.52.0          r36_1     bioconda
bioconductor-bsseq            1.20.0  r36he1b5a44_1     bioconda
```

```
bioconductor-delayedarray 0.10.0          r36h516909a_1    bioconda
bioconductor-delayedmatrixstats 1.6.0                 r36_1    bioconda
bioconductor-geneplotter  1.62.0                  r36_1    bioconda
bioconductor-genomeinfodb 1.20.0                  r36_1    bioconda
bioconductor-genomeinfodbdata 1.2.1                   r36_1    bioconda
bioconductor-genomicalignments 1.20.1          r36h516909a_0    bioconda
bioconductor-genomicranges 1.36.0          r36h516909a_1    bioconda
bioconductor-hdf5array    1.12.1          r36h516909a_0    bioconda
bioconductor-iranges      2.18.1          r36h516909a_0    bioconda
bioconductor-limma        3.40.2          r36h516909a_0    bioconda
bioconductor-methylseekr  1.24.0                  r36_1    bioconda
bioconductor-noiseq       2.28.0                  r36_1    bioconda
bioconductor-rhdf5        2.28.0          r36he1b5a44_1    bioconda
bioconductor-rhdf5lib     1.6.0           r36h516909a_1    bioconda
bioconductor-rhtslib      1.16.1          r36hbcae180_1    bioconda
bioconductor-rsamtools    2.0.0           r36he1b5a44_1    bioconda
bioconductor-rtracklayer  1.44.2          r36h516909a_1    bioconda
bioconductor-s4vectors    0.22.0          r36h516909a_1    bioconda
bioconductor-summarizedexperiment 1.14.0                  r36_1    bioconda
bioconductor-xvector      0.24.0          r36h516909a_1    bioconda
bioconductor-zlibbioc     1.30.0          r36h516909a_1    bioconda
boto3                     1.9.211                  py_0    conda-forge
botocore                  1.12.211                 py_0    conda-forge
bwa                       0.7.17             hed695b0_6    bioconda
bwameth                   0.2.0                 py36_1    bioconda
bwidget                   1.9.11                     0    conda-forge
bzip2                     1.0.8              h516909a_0    conda-forge
ca-certificates           2019.6.16          hecc5488_0    conda-forge
cachetools                2.1.0                    py_0    conda-forge
cairo                     1.16.0          h18b612c_1001    conda-forge
certifi                   2019.6.16             py36_1    conda-forge
cffi                      1.12.3         py36h8022711_0    conda-forge
chardet                   3.0.4              py36_1003    conda-forge
click                     7.0                      py_0    conda-forge
colormath                 3.0.0                    py_2    conda-forge
configargparse            0.13.0                   py_1    conda-forge
cryptography              2.7            py36h72c5cf5_0    conda-forge
curl                      7.65.3             hf8cf82a_0    conda-forge
cycler                    0.10.0                   py_1    conda-forge
datrie                    0.8           py36h516909a_0    conda-forge
dbus                      1.13.6             he372182_0    conda-forge
ddt                       1.2.1                  pypi_0    pypi
decorator                 4.4.0                    py_0    conda-forge
docutils                  0.15.2                py36_0    conda-forge
dropbox                   9.4.0                    py_0    conda-forge
expat                     2.2.5           he1b5a44_1003    conda-forge
fastqc                    0.11.8                     1    bioconda
filechunkio               1.8                      py_2    conda-forge
font-ttf-dejavu-sans-mono 2.37               h6964260_0
fontconfig                2.13.1          he4413a7_1000    conda-forge
freetype                  2.10.0             he983fc9_1    conda-forge
ftputil                   3.4                      py_0    conda-forge
future                    0.17.1              py36_1000    conda-forge
gcc_impl_linux-64         7.3.0              habb00fd_1    conda-forge
gcc_linux-64              7.3.0              h553295d_8    conda-forge
gettext                   0.19.8.1        hc5be6a0_1002    conda-forge
gfortran_impl_linux-64    7.3.0              hdf63c60_1
gfortran_linux-64         7.3.0              h553295d_8
giflib                    5.1.7              h516909a_1    conda-forge
gitdb2                    2.0.5                    py_0    conda-forge
gitpython                 3.0.1                    py_0    conda-forge
glib                      2.58.3          h6f030ca_1002    conda-forge
google-api-core           1.14.2                py36_0    conda-forge
google-auth               1.6.3                    py_0    conda-forge
google-cloud-core         1.0.3                    py_0    conda-forge
google-cloud-storage      1.18.0                   py_0    conda-forge
google-resumable-media    0.3.2                    py_0    conda-forge
googleapis-common-protos  1.6.0                 py36_0    conda-forge
graphite2                 1.3.13          hf484d3e_1000    conda-forge
graphviz                  2.38.0          hf68f40c_1011    conda-forge
gsl                       2.5                h294904e_0    conda-forge
gst-plugins-base          1.14.5             h0935bb2_0    conda-forge
gstreamer                 1.14.5             h36ae1b5_0    conda-forge
gxx_impl_linux-64         7.3.0              hdf63c60_1    conda-forge
gxx_linux-64              7.3.0              h553295d_8    conda-forge
h5py                      2.9.0         nompi_py36h513d04c_1104    conda-forge
harfbuzz                  2.4.0              h37c48d4_1    conda-forge
hdf5                      1.10.5        nompi_h3c11f04_1102    conda-forge
htslib                    1.9                ha228f0b_7    bioconda
icu                       58.2            hf484d3e_1000    conda-forge
idna                      2.8                 py36_1000    conda-forge
idna_ssl                  1.1.0               py36_1000    conda-forge
jinja2                    2.10.1                   py_0    conda-forge
jmespath                  0.9.4                    py_0    conda-forge
jpeg                      9c              h14c3975_1001    conda-forge
```

jsonschema	3.0.2	py36_0	conda-forge
kiwisolver	1.1.0	py36hc9558a2_0	conda-forge
krb5	1.16.3	h05b26f9_1001	conda-forge
lcms2	2.9	h2e4bb80_0	conda-forge
libblas	3.8.0	12_openblas	conda-forge
libcblas	3.8.0	12_openblas	conda-forge
libcurl	7.65.3	hda55be3_0	conda-forge
libdeflate	1.0	h14c3975_1	bioconda
libedit	3.1.20170329	hf8c457e_1001	conda-forge
libffi	3.2.1	he1b5a44_1006	conda-forge
libgcc-ng	9.1.0	hdf63c60_0	
libgfortran-ng	7.3.0	hdf63c60_0	
libiconv	1.15	h516909a_1005	conda-forge
liblapack	3.8.0	12_openblas	conda-forge
libopenblas	0.3.7	h6e990d7_1	conda-forge
libpng	1.6.37	hed695b0_0	conda-forge
libprotobuf	3.9.1	h8b12597_0	conda-forge
libssh2	1.8.2	h22169c7_2	conda-forge
libstdcxx-ng	9.1.0	hdf63c60_0	
libtiff	4.0.10	h57b8799_1003	conda-forge
libtool	2.4.6	h14c3975_1002	conda-forge
libuuid	2.32.1	h14c3975_1000	conda-forge
libxcb	1.13	h14c3975_1002	conda-forge
libxml2	2.9.9	h13577e0_2	conda-forge
lz4-c	1.8.3	he1b5a44_1001	conda-forge
lzstring	1.0.4	py_1001	conda-forge
make	4.2.1	h14c3975_2004	conda-forge
markdown	3.1.1	py_0	conda-forge
markupsafe	1.1.1	py36h14c3975_0	conda-forge
matplotlib	3.1.1	py36_0	conda-forge
matplotlib-base	3.1.1	py36hfd891ef_0	conda-forge
methyldackel	0.4.0	hc0aa232_0	bioconda
metilene	0.2.6	h14c3975_2	bioconda
mosdepth	0.2.5	hb763d49_0	bioconda
multidict	4.5.2	py36h14c3975_1000	conda-forge
multiqc	1.7	py_4	bioconda
ncurses	6.1	hf484d3e_1002	conda-forge
networkx	2.3	py_0	conda-forge
numpy	1.17.0	py36h95a1406_0	conda-forge
openjdk	11.0.1	h46a85a0_1017	conda-forge
openssl	1.1.1c	h516909a_0	conda-forge
pandas	0.25.0	py36hb3f55d8_0	conda-forge
pango	1.40.14	he7ab937_1005	conda-forge
paramiko	2.6.0	py36_0	conda-forge
pcre	8.41	hf484d3e_1003	conda-forge
perl	5.26.2	h516909a_1006	conda-forge
picard	2.20.5	0	bioconda
pip	19.2.2	py36_0	conda-forge
pixman	0.38.0	h516909a_1003	conda-forge
prettytable	0.7.2	py_3	conda-forge
protobuf	3.9.1	py36he1b5a44_0	conda-forge
psutil	5.6.3	py36h516909a_0	conda-forge
pthread-stubs	0.4	h14c3975_1001	conda-forge
pyasn1	0.4.6	py_0	conda-forge
pyasn1-modules	0.2.6	py_0	conda-forge
pycparser	2.19	py36_1	conda-forge
pygments	2.4.2	py_0	conda-forge
pygraphviz	1.5	py36h516909a_1001	conda-forge
pynacl	1.3.0	py36h14c3975_1000	conda-forge
pyopenssl	19.0.0	py36_0	conda-forge
pyparsing	2.4.2	py_0	conda-forge
pyqt	5.9.2	py36hcca6a23_2	conda-forge
pyrsistent	0.15.4	py36h516909a_0	conda-forge
pysam	0.15.3	py36hda2845c_1	bioconda
pysftp	0.2.9	py_1	conda-forge
pysocks	1.7.0	py36_0	conda-forge
python	3.6.7	h357f687_1005	conda-forge
python-dateutil	2.8.0	py_0	conda-forge
python-irodsclient	0.7.0	py_0	conda-forge
pytz	2019.2	py_0	conda-forge
pyyaml	5.1.2	py36h516909a_0	conda-forge
qt	5.9.7	h52cfd70_2	conda-forge
qualimap	2.2.2c	1	bioconda
r-assertthat	0.2.1	r36h6115d3f_1	conda-forge
r-backports	1.1.4	r36hcdcec82_1	conda-forge
r-base	3.6.1	h8900bf8_2	conda-forge
r-bh	1.69.0_1	r36h6115d3f_1	conda-forge
r-bit	1.1_14	r36hcdcec82_1	conda-forge
r-bit64	0.9_7	r36hcdcec82_1001	conda-forge
r-bitops	1.0_6	r36hcdcec82_1003	conda-forge
r-blob	1.2.0	r36_1	conda-forge
r-cli	1.1.0	r36h6115d3f_1	conda-forge
r-colorspace	1.4_1	r36hcdcec82_1	conda-forge
r-crayon	1.3.4	r36h6115d3f_1002	conda-forge
r-crosstalk	1.0.0	r36h6115d3f_1002	conda-forge

r−data.table	1.12.2	r36hcdcec82_1	conda−forge
r−dbi	1.0.0	r36h6115d3f_1002	conda−forge
r−digest	0.6.20	r36h0357c0b_1	conda−forge
r−dt	0.8	r36h6115d3f_0	conda−forge
r−ellipsis	0.2.0.1	r36hcdcec82_1	conda−forge
r−fansi	0.4.0	r36hcdcec82_1001	conda−forge
r−formatr	1.7	r36h6115d3f_1	conda−forge
r−futile.logger	1.4.3	r36h6115d3f_1002	conda−forge
r−futile.options	1.0.1	r36h6115d3f_1001	conda−forge
r−getopt	1.20.3	r36_1	conda−forge
r−ggplot2	3.2.1	r36h6115d3f_0	conda−forge
r−glue	1.3.1	r36hcdcec82_1	conda−forge
r−gridextra	2.3	r36h6115d3f_1002	conda−forge
r−gtable	0.3.0	r36h6115d3f_2	conda−forge
r−gtools	3.8.1	r36hcdcec82_1003	conda−forge
r−htmltools	0.3.6	r36he1b5a44_1003	conda−forge
r−htmlwidgets	1.3	r36h6115d3f_1001	conda−forge
r−httpuv	1.5.1	r36h0357c0b_1	conda−forge
r−jsonlite	1.6	r36hcdcec82_1001	conda−forge
r−labeling	0.3	r36h6115d3f_1002	conda−forge
r−lambda.r	1.2.3	r36h6115d3f_1001	conda−forge
r−later	0.8.0	r36h0357c0b_1	conda−forge
r−lattice	0.20_38	r36hcdcec82_1002	conda−forge
r−lazyeval	0.2.2	r36hcdcec82_1	conda−forge
r−locfit	1.5_9.1	r36h516909a_1004	conda−forge
r−magrittr	1.5	r36h6115d3f_1002	conda−forge
r−mass	7.3_51.4	r36hcdcec82_1	conda−forge
r−matrix	1.2_17	r36hcdcec82_1	conda−forge
r−matrixstats	0.54.0	r36hcdcec82_1001	conda−forge
r−memoise	1.1.0	r36h6115d3f_1002	conda−forge
r−mgcv	1.8_28	r36hcdcec82_1	conda−forge
r−mhsmm	0.4.16	r36h516909a_1003	conda−forge
r−mime	0.7	r36hcdcec82_1	conda−forge
r−munsell	0.5.0	r36h6115d3f_1002	conda−forge
r−mvtnorm	1.0_11	r36h9bbef5b_1	conda−forge
r−nlme	3.1_141	r36h9bbef5b_1	conda−forge
r−optparse	1.6.2	r36h6115d3f_1	conda−forge
r−permute	0.9_5	r36_1	conda−forge
r−pillar	1.4.2	r36h6115d3f_2	conda−forge
r−pkgconfig	2.0.2	r36h6115d3f_1002	conda−forge
r−plogr	0.2.0	r36h6115d3f_1002	conda−forge
r−plyr	1.8.4	r36h0357c0b_1003	conda−forge
r−prettyunits	1.0.2	r36h6115d3f_1002	conda−forge
r−promises	1.0.1	r36h0357c0b_1001	conda−forge
r−r.methodss3	1.7.1	r36h6115d3f_1002	conda−forge
r−r.oo	1.22.0	r36h6115d3f_1001	conda−forge
r−r.utils	2.9.0	r36h6115d3f_1	conda−forge
r−r6	2.4.0	r36h6115d3f_2	conda−forge
r−rcolorbrewer	1.1_2	r36h6115d3f_1002	conda−forge
r−rcpp	1.0.2	r36h0357c0b_0	conda−forge
r−rcurl	1.95_4.12	r36hcdcec82_1	conda−forge
r−reshape2	1.4.3	r36h0357c0b_1004	conda−forge
r−rlang	0.4.0	r36hcdcec82_1	conda−forge
r−rsqlite	2.1.2	r36h0357c0b_0	conda−forge
r−scales	1.0.0	r36h0357c0b_1002	conda−forge
r−shiny	1.3.2	r36h6115d3f_1	conda−forge
r−shinydashboard	0.7.1	r36h6115d3f_1001	conda−forge
r−snow	0.4_3	r36h6115d3f_1001	conda−forge
r−sourcetools	0.1.7	r36he1b5a44_1001	conda−forge
r−stringi	1.4.3	r36h0357c0b_2	conda−forge
r−stringr	1.4.0	r36h6115d3f_1	conda−forge
r−tibble	2.1.3	r36hcdcec82_1	conda−forge
r−upsetr	1.4.0	r36h6115d3f_1	conda−forge
r−utf8	1.1.4	r36hcdcec82_1001	conda−forge
r−vctrs	0.2.0	r36hcdcec82_1	conda−forge
r−viridislite	0.3.0	r36h6115d3f_1002	conda−forge
r−withr	2.1.2	r36h6115d3f_1001	conda−forge
r−xml	3.98_1.20	r36hcdcec82_1	conda−forge
r−xtable	1.8_4	r36h6115d3f_2	conda−forge
r−yaml	2.2.0	r36hcdcec82_1002	conda−forge
r−zeallot	0.1.0	r36h6115d3f_1001	conda−forge
ratelimiter	1.2.0	py36_1000	conda−forge
readline	8.0	hf8c457e_0	conda−forge
requests	2.22.0	py36_1	conda−forge
rsa	3.4.2	py_1	conda−forge
ruamel−yaml	0.16.5	pypi_0	pypi
ruamel−yaml−clib	0.1.2	pypi_0	pypi
s3transfer	0.2.1	py36_0	conda−forge
samtools	1.9	h8571acd_11	bioconda
sed	4.7	h1bed415_1000	conda−forge
setuptools	41.0.1	py36_0	conda−forge
simplejson	3.16.1	py36h470a237_0	conda−forge
sip	4.19.8	py36hf484d3e_1000	conda−forge
six	1.12.0	py36_1000	conda−forge
smmap2	2.0.5	py_0	conda−forge

snakemake	5.5.4	1	bioconda
snakemake-minimal	5.5.4	py_1	bioconda
spectra	0.0.11	py_1	conda-forge
sqlite	3.29.0	hcee41ef_0	conda-forge
tk	8.6.9	hed695b0_1002	conda-forge
tktable	2.10	h555a92e_1	conda-forge
toolshed	0.4.6	py_1	bioconda
tornado	6.0.3	py36h516909a_0	conda-forge
typing_extensions	3.7.4	py36_0	conda-forge
urllib3	1.25.3	py36_0	conda-forge
wg-blimp	0.9.3	dev_0	<develop>
wheel	0.33.6	py36_0	conda-forge
wrapt	1.11.2	py36h516909a_0	conda-forge
xmlrunner	1.7.7	py_0	conda-forge
xorg-fixesproto	5.0	h14c3975_1002	conda-forge
xorg-inputproto	2.3.2	h14c3975_1002	conda-forge
xorg-kbproto	1.0.7	h14c3975_1002	conda-forge
xorg-libice	1.0.10	h516909a_0	conda-forge
xorg-libsm	1.2.3	h84519dc_1000	conda-forge
xorg-libx11	1.6.8	h516909a_0	conda-forge
xorg-libxau	1.0.9	h14c3975_0	conda-forge
xorg-libxdmcp	1.1.3	h516909a_0	conda-forge
xorg-libxext	1.3.4	h516909a_0	conda-forge
xorg-libxfixes	5.0.3	h516909a_1004	conda-forge
xorg-libxi	1.7.10	h516909a_0	conda-forge
xorg-libxpm	3.5.12	h14c3975_1002	conda-forge
xorg-libxrender	0.9.10	h516909a_1002	conda-forge
xorg-libxt	1.1.5	h516909a_1003	conda-forge
xorg-libxtst	1.2.3	h14c3975_1002	conda-forge
xorg-recordproto	1.14.2	h14c3975_1002	conda-forge
xorg-renderproto	0.11.1	h14c3975_1002	conda-forge
xorg-xextproto	7.3.0	h14c3975_1002	conda-forge
xorg-xproto	7.0.31	h14c3975_1007	conda-forge
xz	5.2.4	h14c3975_1001	conda-forge
yaml	0.1.7	h14c3975_1001	conda-forge
yarl	1.3.0	py36h14c3975_1000	conda-forge
zlib	1.2.11	h516909a_1005	conda-forge
zstd	1.4.0	h3b9ef0a_0	conda-forge

3 Run time benchmark of published WGBS pipelines

We applied the pipelines listed in Table 1 to the blood/sperm dataset (ENA accession PRJEB28044) to compare run times across different workflows. For each workflow we followed the guidelines given by the respective authors and used a setup as close to the default settings as possible. As each pipeline has a unique setup, we provide detailed descriptions about the setup used for each analysis.

All analyses (except the CpG_Me workflow, see subsection 3.3 for details) were executed on servers equipped with two Intel Xeon E5-2695 v4 CPU's, 528 GB of memory and Debian 9 as operating system (OS). For each workflow we assigned a maximum of 64 cores and limited alignment jobs to a maximum of 16 cores where possible. This speeds up execution because all four samples can be processed concurrently, thus minimizing run time owed to sequential portions of alignment programs. We ensured each pipeline had exclusive access to server resources to prevent other processes from interfering with benchmark results. In addition, all computations were performed on a partition excluded from backups to prevent our backup processes from slowing down pipeline runs. All pipelines (except Methy-Pipe, see subsection 3.4 for details) used hg38 as reference. We excluded installation and reference index generation run times as these operations only need to be executed once before potentially analysing multiple datasets.

While we encountered technical issues with several pipelines, we still report each pipeline's run time until failure in cases where the remaining computations would unlikely have caused substantial computational overhead. For example, DMR calling requires only a fraction of computational resources of alignment and methylation calling. While we can not entirely

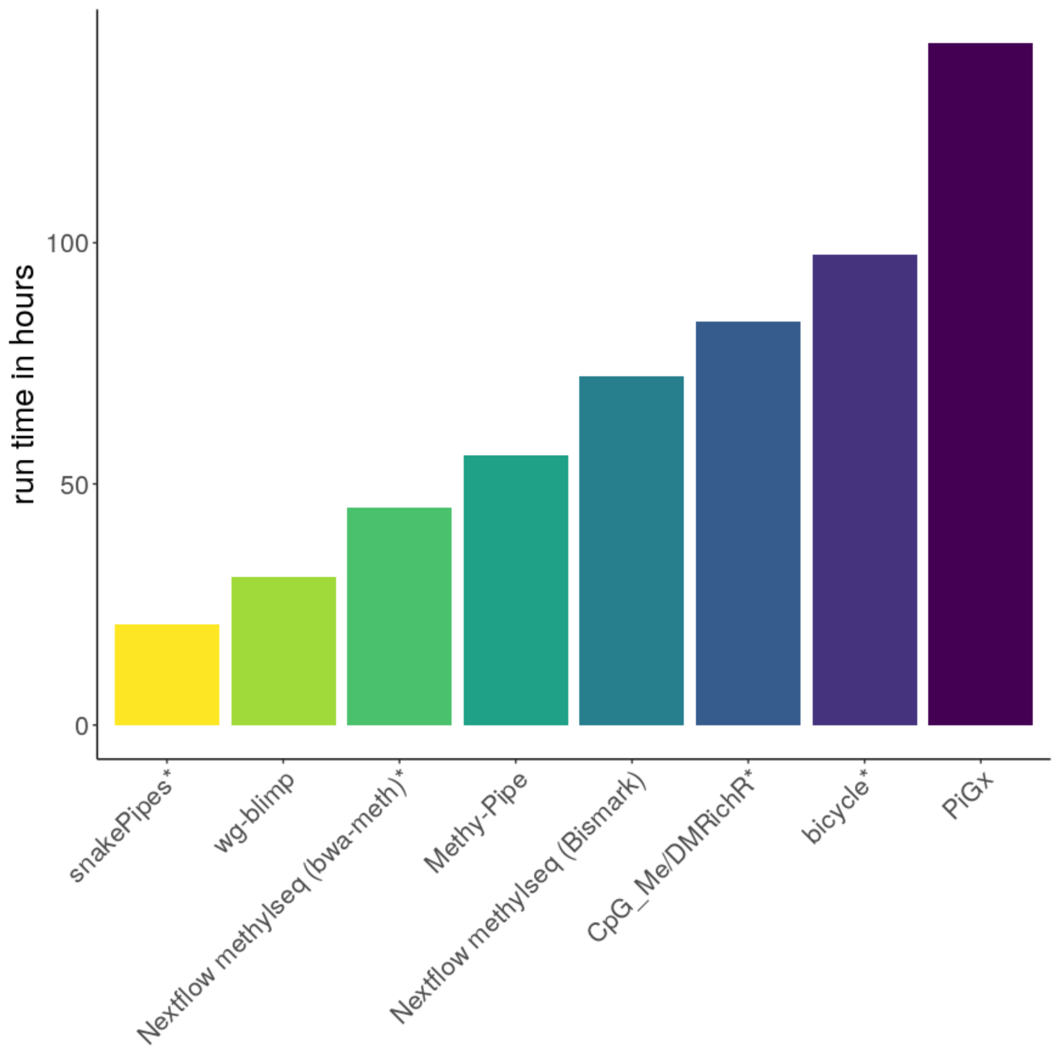

Figure S13: Run times of pipelines for blood/sperm dataset. Pipelines marked with an asterisk (*) showed technical issues when analysing the dataset.

rule out technical issues originating from our misuse of the respective pipelines, we attempted to be as exhaustive as possible when searching for the causes of these errors. Furthermore, some of these issues may already be known to the developers and will be fixed in the near future, as several pipelines are under active development. Nevertheless, we excluded BAT from the analysis because it processed only a small fraction of raw reads (see subsection 3.1 for details). We also excluded the ENCODE pipeline because we have no adequate control over the benchmark environment hosted at DNAnexus.

We note that such a benchmark has several caveats and is only intended to provide information about the order of magnitude of each pipeline's run time for a WGBS dataset: The technical issues prevent getting exact run times and may result in underestimation of

pipeline run times in cases of failure. Also, such a comparison may disfavour feature-rich pipelines, as more features included in a workflow will inherently increase its run time. Similarly, tools may not primarily be selected for their performance, but their stability and precision.

Figure S13 shows the run times of all pipelines for the blood/sperm dataset. Pipelines built on top of bwa-meth/MethylDackel (Nextflow methylseq (bwa-meth), snakePipes, wg-blimp) show the fastest execution times, whereas pipelines utilizing Bismark (CpG_Me/DMRichR, Nextflow methylseq (Bismark), PiGx) require more time to complete. Although wg-blimp shows the second lowest run-time, it should be noted that it was developed and optimised to be run on our local infrastructure and has an inherent performance advantage as a result.

We conclude that all pipelines show adequate run times for the blood/sperm dataset, with wg-blimp performing similar as other bwa-meth based workflows. However, we note that for higher sample counts (hundreds or even thousands of samples) we would prefer one of the workflows based on bwa-meth to minimize processing times (Nextflow methylseq (bwa-meth), snakePipes or wg-blimp).

The following sections describe configurations and scripts used for each pipeline run. A detailed description of wg-blimp's configuration for this dataset can be found in subsection 2.5.

3.1 BAT configuration

We executed BAT using the Docker container christianbioinf/bat (digest 51f4d7aa7104) and the following script:

```
PATH=$PATH:$(pwd)

###########
# mapping #
###########
echo "Mapping Module"
# mapping

RAW_FASTQ=/raw/
SAMPLES="60_Hmp01_blood_young 60_Hmp02_blood_old 60_Hmp01_sperm_young 60_Hmp02_sperm_old"
FORWARD_SUFFIX=_R1.fastq.gz
REVERSE_SUFFIX=_R2.fastq.gz

GROUP1="60_Hmp01_blood_young,60_Hmp02_blood_old"
GROUP2="60_Hmp01_sperm_young,60_Hmp02_sperm_old"

GENES=/reference/genes.bed
REFERENCE_PREFIX=/reference/assembled
FASTA_FILE=$REFERENCE_PREFIX.fa

THREADS=64

for SAMPLE in $SAMPLES; do

  BAT_mapping -g $FASTA_FILE -q $RAW_FASTQ/$SAMPLE$FORWARD_SUFFIX -p $RAW_FASTQ/
      ↪ $SAMPLE$REVERSE_SUFFIX -i $REFERENCE_PREFIX -o mapped/$SAMPLE -t $THREADS

  BAT_mapping_stat --bam mapped/$SAMPLE.bam --excluded mapped/$SAMPLE.excluded.bam --fastq
      ↪ $RAW_FASTQ/$SAMPLE$FORWARD_SUFFIX >mapped/$SAMPLE.stat
  BAT_calling -d $FASTA_FILE -q mapped/$SAMPLE.bam -o called/

  BAT_filter_vcf --vcf called/$SAMPLE.vcf.gz --out called/$SAMPLE\_CG.vcf.gz --context CG --
      ↪ MDP_min 10 --MDP_max 100;
done


############
# analysis #
############
echo "Analysis Module"
mkdir -p DMRs
```

```
# summarize
BAT_summarize --in1 $(echo called/{60_Hmp01_blood_young,60_Hmp02_blood_old}_CG.bedgraph | tr ' '
    ↪ ,) --in2 $(echo called/{60_Hmp01_sperm_young,60_Hmp02_sperm_old}_CG.bedgraph | tr ' ' ,)
    ↪ --groups control,case --h1 $GROUP1 --h2 $GROUP2 --out example  --cs $FASTA_FILE.sizes


# overview
BAT_overview.R  -i example_summary_control_case.bedgraph -o example_overview --groups control,
    ↪ case


########
# DMRs #
########
echo "DMR Module"
# DMR calling
BAT_DMRcalling -q example_metilene_control_case.txt -o DMRs/metilene -a control -b case

# cDMRs
bedtools intersect -wa -wb -a DMRs/metilene_qval.0.05.bed -b $GENES | cut -f1-3,9 >DMRs/DMR_gene.
    ↪ txt
ls *.bw | grep -v "mean" | grep -v "diff" | sed 's/\t/\n/' > DMRs/methylation_files.list

# omitted: correlation and annotation
```

We noticed BAT completed its run after a short run time with only a fraction of raw reads processed. We think the cause of this issue is not BAT itself, but its internal usage of segemehl. When manually executing the command

```
segemehl.x -s -d /reference/assembled.fa -i /reference/assembled.ctidx -j /reference/assembled.
    ↪ gaidx -F 1 -t 64 -q /raw/60_Hmp01_blood_young_R1.fastq.gz -u /data/mapped/60
    ↪ _Hmp01_blood_young.unmapped -o /data/mapped/60_Hmp01_blood_young.sam -p /raw/60
    ↪ _Hmp01_blood_young_R2.fastq.gz
```

it reports

```
...
[SEGEMEHL] Wed Jan  8 12:57:47 2020: reading queries in '/raw/60_Hmp01_blood_young_R1.fastq.gz'.
[SEGEMEHL] Wed Jan  8 12:59:32 2020: 20000000 query sequences found.
[SEGEMEHL] Wed Jan  8 12:59:32 2020: reading mates in '/raw/60_Hmp01_blood_young_R2.fastq.gz'.
[SEGEMEHL] Wed Jan  8 13:01:11 2020: 20000000 mate sequences found.
...
```

before completion. We speculate that segemehl has an upper limit of reads it can process in a single run. While this causes no issues when many input files with low read counts are provided, it is discarding most of the reads when fed single files with high read counts. As this issue drastically distorts BAT's run time for the blood/sperm dataset, we omit it from the comparison.

3.2 bicycle configuration

We ran bicycle using the docker container singgroup/bicycle:1.8.1 (digest a26ca05ce4d3). The following script was used for running bicycle:

```
#!/bin/bash

RESULTS_DIR=results
REFERENCE_DIR=/media/holmes/genomes/Homo_sapiens.GRCh38.p7/
READ_ROOT_FOLDER=/media/holmes/backup-exclude/wg-blimp-eval-blood-sperm-pipes/fastq
READS_PER_SAMPLE_DIR=fastq/
R1_REGEX=_R1.fastq
R2_REGEX=_R2.fastq
THREADS=64

GROUP1="60_Hmp01_blood_young,60_Hmp02_blood_old"
GROUP2="60_Hmp01_sperm_young,60_Hmp02_sperm_old"

export _JAVA_OPTIONS="-Xmx64G"

for FILE in $READ_ROOT_FOLDER/*$R1_REGEX; do

  SAMPLE_NAME=$(basename $FILE $R1_REGEX)

  SAMPLE_FASTQ_DIR=$READS_PER_SAMPLE_DIR/$SAMPLE_NAME/
```

```
  mkdir -p $SAMPLE_FASTQ_DIR

  ln -s $READ_ROOT_FOLDER/$SAMPLE_NAME$R1_REGEX $SAMPLE_FASTQ_DIR/$SAMPLE_NAME$R1_REGEX
  ln -s $READ_ROOT_FOLDER/$SAMPLE_NAME$R2_REGEX $SAMPLE_FASTQ_DIR/$SAMPLE_NAME$R2_REGEX
done


bicycle create-project -p $RESULTS_DIR -r $REFERENCE_DIR -f $READS_PER_SAMPLE_DIR

bicycle align --bowtie2-quals phred33 --bowtie2-I 0 --bowtie2-X 500 -p $RESULTS_DIR -t $THREADS

bicycle analyze-methylation -p $RESULTS_DIR -n $THREADS

bicycle analyze-differential-methylation -p $RESULTS_DIR -t $GROUP1 -c $GROUP2
```

Inspecting bicycle's log revealed multiple segmentations faults when running the analysis. In total, bicycle threads produced 264 log files for alignment (66 per sample). 33 of these log files reported core dumps, resulting in expected 12.5% data loss. To ensure we were not simply using bicycle incorrectly, we started the script above using only 2 threads instead of 64. This run did not cause any core dumps, so we suspect bicycle's multithreading implementation to have caused the issue.

3.3 CpG_Me configuration

For CpG_Me/DMRichR we used the code from the official GitHub repository (https://github.com/benlaufer/CpG_Me) at git tag 1.2. A distinct difference between CpG_Me and other pipelines is its reliance on a Slurm execution system. As a result, we could not use our local server infrastructure for a benchmark, but instead used the PALMAII HPC system of the University of Münster. We note that the resources requested by CpG_Me are similar to the requirements of other pipelines described here and we estimate run times to be comparable as a result. While run time measurements may be inaccurate when job submission queues are full, we observed only ∼50% of the 504 nodes being busy during the benchmark using the clusters' Ganglia monitoring system. Since CpG_Me requires only fastq files following strict naming patterns as input, explicit configuration is not needed and not reported here as a result. We invoked the pipeline using the command:

```
sbatch --array=1-4 /scratch/tmp/m_woes11/cpgme/programs/CpG_Me/Paired-end/CpG_Me_PE_controller.sh
    ↪ hg38
```

We could not execute DMRichR as two methylation reports resulted in core dumps, and DMRichR requires at least two samples per group:

```
...
/var/spool/slurm/ctld/job4788244/slurm_script: line 217: 143528 Bus error              (core
    ↪ dumped) coverage2cytosine --output /scratch/tmp/m_woes11/cpgme/Project/60
    ↪ _Hmp01_blood_young/60_Hmp01_blood_young_1_val_1_bismark_bt2_pe.deduplicated.bismark.cov.
    ↪ gz --genome_folder /scratch/tmp/m_woes11/cpgme/genomes/hg38/ --gzip --merge_CpG /scratch/
    ↪ tmp/m_woes11/cpgme/Project/60_Hmp01_blood_young/60
    ↪ _Hmp01_blood_young_1_val_1_bismark_bt2_pe.deduplicated.bismark.cov.gz
...
/var/spool/slurm/ctld/job4788245/slurm_script: line 217: 204597 Bus error              (core
    ↪ dumped) coverage2cytosine --output /scratch/tmp/m_woes11/cpgme/Project/60_Hmp02_sperm_old
    ↪ /60_Hmp02_sperm_old_1_val_1_bismark_bt2_pe.deduplicated.bismark.cov.gz --genome_folder /
    ↪ scratch/tmp/m_woes11/cpgme/genomes/hg38/ --gzip --merge_CpG /scratch/tmp/m_woes11/cpgme/
    ↪ Project/60_Hmp02_sperm_old/60_Hmp02_sperm_old_1_val_1_bismark_bt2_pe.deduplicated.bismark
    ↪ .cov.gz
...
```

We are unsure why this error is occurring, as debugging applications within Slurm is more cumbersome than on our local infrastructure. We are reporting here the conda configuration used by Slurm jobs for potential debugging purposes:

```
# packages in environment at /scratch/tmp/m_woes11/cpgme/conda/envs/cpgme:
#
# Name                    Version                   Build  Channel
_libgcc_mutex             0.1                        main
backports                 1.0                        py_2
backports.functools_lru_cache 1.6.1                  py_0
backports_abc             0.5                      py27_0
bismark                   0.22.3                        0    bioconda
blas                      1.0                         mkl
bowtie                    1.2.3            py27hc9558a2_0    bioconda
bowtie2                   2.3.5            py27he860b03_0    bioconda
bz2file                   0.98                     py27_1
bzip2                     1.0.8                h7b6447c_0
ca-certificates           2019.11.27                    0
certifi                   2019.11.28               py27_0
click                     7.0                      py27_0
curl                      7.67.0               hbc83047_0
cutadapt                  1.18             py27h14c3975_1    bioconda
cycler                    0.10.0                   py27_0
dbus                      1.13.12              h746ee38_0
expat                     2.2.6                he6710b0_0
fastq-screen              0.13.0                  pl526_1    bioconda
fastqc                    0.11.8                        2    bioconda
font-ttf-dejavu-sans-mono 2.37                 h6964260_0
fontconfig                2.13.0               h9420a91_0
freetype                  2.9.1                h8a8886c_1
functools32               3.2.3.2                  py27_1
futures                   3.3.0                    py27_0
giflib                    5.1.4                h14c3975_1
git                       2.23.0          pl526hacde149_0
glib                      2.63.1               h5a9c865_0
gst-plugins-base          1.14.0               hbbd80ab_1
gstreamer                 1.14.0               hb453b48_1
hisat2                    2.1.0            py27hc9558a2_4    bioconda
htslib                    1.9                  h4da6232_3    bioconda
icu                       58.2                 h9c2bf20_1
intel-openmp              2019.4                      243
jinja2                    2.10.3                     py_0
jpeg                      9b                   h024ee3a_2
kiwisolver                1.1.0            py27he6710b0_0
krb5                      1.16.4               h173b8e3_0
libcurl                   7.67.0               h20c2e04_0
libdeflate                1.2                  h516909a_1    bioconda
libedit                   3.1.20181209         hc058e9b_0
libffi                    3.2.1                hd88cf55_4
libgcc-ng                 9.1.0                hdf63c60_0
libgd                     2.2.5                hceca4fd_3
libgfortran-ng            7.3.0                hdf63c60_0
libpng                    1.6.37               hbc83047_0
libssh2                   1.8.2                h1ba5d50_0
libstdcxx-ng              9.1.0                hdf63c60_0
libtiff                   4.1.0                h2733197_0
libuuid                   1.0.3                h1bed415_2
libwebp                   1.0.1                h8e7db2f_0
libxcb                    1.13                 h1bed415_1
libxml2                   2.9.9                hea5a465_1
markupsafe                1.1.1            py27h7b6447c_0
matplotlib                2.2.3            py27hb69df0a_0
mkl                       2019.4                      243
mkl-service               2.3.0            py27he904b0f_0
mkl_fft                   1.0.15           py27ha843d7b_0
mkl_random                1.1.0            py27hd6b4f25_0
multiqc                   1.0.dev0                 pypi_0    pypi
ncurses                   6.1                  he6710b0_1
numpy                     1.16.5           py27h7e9f1db_0
numpy-base                1.16.5           py27hde5b4d6_0
openjdk                   8.0.152              h46b5887_1
openssl                   1.1.1d               h7b6447c_3
pcre                      8.43                 he6710b0_0
perl                      5.26.2               h14c3975_0
perl-gd                   2.71            pl526he860b03_0    bioconda
perl-gdgraph              1.54                    pl526_0    bioconda
perl-gdtextutil           0.86            pl526h14c3975_5    bioconda
pigz                      2.4                  h84994c4_0
pip                       19.3.1                   py27_0
pyparsing                 2.4.6                      py_0
pyqt                      5.9.2            py27h05f1152_2
python                    2.7.17               h9bab390_0
python-dateutil           2.8.1                      py_0
pytz                      2019.3                     py_0
pyyaml                    5.2              py27h7b6447c_0
qt                        5.9.7                h5867ecd_1
readline                  7.0                  h7b6447c_5
samtools                  1.9                 h10a08f8_12    bioconda
setuptools                44.0.0                   py27_0
```

```
simplejson                3.8.1                  py27_0     bioconda
singledispatch            3.4.0.3                py27_0
sip                       4.19.8       py27hf484d3e_0
six                       1.13.0                 py27_0
sqlite                    3.30.1             h7b6447c_0
subprocess32              3.5.4        py27h7b6447c_0
tbb                       2019.8             hfd86e86_0
tk                        8.6.8              hbc83047_0
tornado                   5.1.1        py27h7b6447c_0
trim-galore               0.6.5                       0     bioconda
wheel                     0.33.6                 py27_0
xopen                     0.7.3                    py_0     bioconda
xz                        5.2.4              h14c3975_4
yaml                      0.1.7              had09818_2
zlib                      1.2.11             h7b6447c_3
zstd                      1.3.7              h0b5b093_0
```

3.4 Methy-Pipe configuration

We manually installed perl 5.26.2 and R 3.6.2 within a conda installation to run Methy-Pipe. Methy-Pipe 2.02 was downloaded from the official website at https://sunlab.cpy.cuhk.edu.hk/methy-pipe/ . We have been unable to run Methy-Pipe for hg38 as there is few documentation regarding usage of non-default reference genomes. As a result, we used its default genome configuration that is bundled with the software download. The Makefile Methy-Pipe uses for analysis was generated according to the examples provided with the software. The following configuration was used:

```
# standard configuration
# statistics program R. please install ggplot2 (http://ggplot2.org/)
#R       /usr/local/bin/R
R        /media/watson/scripts/marw-conda/envs/methypipe/bin/R
# BS-seq aligner
BSAligner        ../methy-pipe2/cpp_prog/BSAligner
#reference genome index for the BSAaligner
BS_INDEX         ../methy-pipe2/database/hg19
#each chromosome length
LIST_CHR_LEN     ../methy-pipe2/database/hg19.size
#Watson strand reference (fasta)
GENOME_W_FA      ../methy-pipe2/database/hg19.W.ori.fa
#Crick strand reference (fasta)
GENOME_C_FA      ../methy-pipe2/database/hg19.C.ori.fa
#frequency for each 3mer in reference genome
HG_3MER ../methy-pipe2/database/hg19.3mer
#windows around TSS (ucsc reference gene)
TSS      ../methy-pipe2/database/TSS.win.bed
#sequencing data format fastq (fq) or fasta (fa)
SEQ_FORMAT       fq
#prefix for each output result
OUT_PREFIX       results
#sequencing mode in a paired-end manner (PE) or single-end manner (SE)
SEQ_MODE        PE
#how many first cycles supposed to be used, for example, 75 means the cycles after 75th would be
    ↪ omitted
USED_CYCLES      100
#how many threads supposed to be used for the BSAligner
THREAD   64
#whether to merge the all of alignments in this run
MERGE    0
#window size to profile the methylation density across the genome when considering the CpG sites
BIN_SIZE_CPG     100e3
#window size to profile the methylation density across the genome when considering the non-CpG
    ↪ sites
BIN_SIZE_NONCPG 100e3
#how many total cycles supposed to be used (read1+read2).
SEQUENC_TOT_CYCLE        200
#a separated files recording the path of fastq or fasta as well as the sample names that are to
    ↪ be analyzed
INFO     ./info
#the output directory
OUT_DIR Methy-Pipe2_output
#mismatch allowed for each end
MISMATCH         2
#minimal insert size allowed for paired-end reads
MIN_INS 0
#maximal insert size allowed for paired-end reads
MAX_INS 600
```

The info file used to identify samples looks as follows:

```
#sample-id	lane-number	sample-descriptions	path-to-raw-fastq-data
60_Hmp01_blood_young	1	60_Hmp01_blood_young	/media/holmes/backup-exclude/wg-blimp-
    ↪ eval-blood-sperm-pipes/fastq/60_Hmp01_blood_young_R1.fastq	/media/holmes/backup-
    ↪ exclude/wg-blimp-eval-blood-sperm-pipes/fastq/60_Hmp01_blood_young_R2.fastq
60_Hmp02_blood_old	2	60_Hmp02_blood_old	/media/holmes/backup-exclude/wg-blimp-
    ↪ eval-blood-sperm-pipes/fastq/60_Hmp02_blood_old_R1.fastq	/media/holmes/backup-exclude/wg
    ↪ -blimp-eval-blood-sperm-pipes/fastq/60_Hmp02_blood_old_R2.fastq
60_Hmp01_sperm_young	3	60_Hmp01_sperm_young	/media/holmes/backup-exclude/wg-blimp-
    ↪ eval-blood-sperm-pipes/fastq/60_Hmp01_sperm_young_R1.fastq	/media/holmes/backup-
    ↪ exclude/wg-blimp-eval-blood-sperm-pipes/fastq/60_Hmp01_sperm_young_R2.fastq
60_Hmp02_sperm_old	4	60_Hmp02_sperm_old	/media/holmes/backup-exclude/wg-blimp-
    ↪ eval-blood-sperm-pipes/fastq/60_Hmp02_sperm_old_R1.fastq	/media/holmes/backup-exclude/wg
    ↪ -blimp-eval-blood-sperm-pipes/fastq/60_Hmp02_sperm_old_R2.fastq
```

3.5 Nextflow methylseq (Bismark) configuration

For Nextflow methylseq (Bismark) we used the following configuration (as reported by Nextflow):

```
----------------------------------------------------
                                        ,--./,-.
        ___     __   __   __   ___     /,-._.--~\
  |\ | |__  __ /  ` /  \ |__) |__         }  {
  | \| |       \__, \__/ |  \ |___     \`-._,-`-,
                                        `._,._,'
  nf-core/methylseq v1.4
----------------------------------------------------

Run Name: lethal_curran

## nf-core/methylseq execution completed successfully! ##

The workflow was completed at 2020-01-19T14:48:58.088595+01:00 (duration: 3d 20h 21m 10s)

The command used to launch the workflow was as follows:

  nextflow run nf-core/methylseq -profile docker --reads '/media/holmes/backup-exclude/wg-blimp-
      ↪ eval-blood-sperm-pipes/fastq/*_R{1,2}.fastq.gz' --fasta /media/holmes/genomes/
      ↪ Homo_sapiens.GRCh38.p7/assembled.fa --bismark_index /media/holmes/genomes/Homo_sapiens.
      ↪ GRCh38.p7/ --fasta_index /media/holmes/genomes/Homo_sapiens.GRCh38.p7/assembled.fa.fai
      ↪ --max_cpus 64 --max_time 2400.h



Pipeline Configuration:
-----------------------
 - Pipeline Release: master
 - Pipeline Name: nf-core/methylseq
 - Run Name: lethal_curran
 - Reads: /media/holmes/backup-exclude/wg-blimp-eval-blood-sperm-pipes/fastq/*_R{1,2}.fastq.gz
 - Aligner: bismark
 - Spliced alignment: No
 - SLAM-seq: No
 - Local alignment: No
 - Data Type: Paired-End
 - Genome: false
 - Bismark Index: /media/holmes/genomes/Homo_sapiens.GRCh38.p7/
 - Fasta Ref: /media/holmes/genomes/Homo_sapiens.GRCh38.p7/assembled.fa
 - Fasta Index: /media/holmes/genomes/Homo_sapiens.GRCh38.p7/assembled.fa.fai
 - Trim R1: 0
 - Trim R2: 0
 - Trim 3' R1: 0
 - Trim 3' R2: 0
 - Deduplication: Yes
 - Directional Mode: Yes
 - All C Contexts: No
 - Save Reference: No
 - Save Trimmed: No
 - Save Unmapped: No
 - Save Intermediates: No
 - Current home: /home/marw
 - Current path: /media/holmes/backup-exclude/wg-blimp-eval-blood-sperm-pipes/nextflow_bismark
 - Max Resources: 128 GB memory, 64 cpus, 2400.h time per job
 - Container: docker - nfcore/methylseq:1.4
 - Output dir: ./results
 - Launch dir: /media/holmes/backup-exclude/wg-blimp-eval-blood-sperm-pipes/nextflow_bismark
 - Working dir: /media/holmes/backup-exclude/wg-blimp-eval-blood-sperm-pipes/nextflow_bismark/
     ↪ work
 - Script dir: /home/marw/.nextflow/assets/nf-core/methylseq
```

```
 - User: marw
 - Config Profile: docker
 - Date Started: 2020-01-15T18:27:47.680940+01:00
 - Date Completed: 2020-01-19T14:48:58.088595+01:00
 - Pipeline script file path: /home/marw/.nextflow/assets/nf-core/methylseq/main.nf
 - Pipeline script hash ID: f6350c2414e08a0f04d816270b4d22dc
 - Pipeline repository Git URL: https://github.com/nf-core/methylseq.git
 - Pipeline repository Git Commit: 40760ecffbf1d3ce8659712f692454ed88e59324
 - Pipeline Git branch/tag: master
 - Nextflow Version: 19.10.0
 - Nextflow Build: 5170
 - Nextflow Compile Timestamp: 21-10-2019 15:07 UTC

--
nf-core/methylseq
https://github.com/nf-core/methylseq
```

Please note that we intentionally set the maximum run time per job to 2 400 hours to prevent pipeline timeouts from extending run time.

3.6 Nextflow methylseq (bwa-meth) configuration

The configuration for this workflow is similar to the Bismark configuration above:

```
----------------------------------------------------
                                        ,--./,-.
        ___     __   __   __   ___     /,-._.--~\
  |\ | |__  __ /  ` /  \ |__) |__         }  {
  | \| |       \__, \__/ |  \ |___     \`-._,-`-,
                                        `._,._,'
  nf-core/methylseq v1.4
----------------------------------------------------

Run Name: berserk_celsius

## nf-core/methylseq execution completed successfully! ##

The workflow was completed at 2020-01-22T07:16:29.439988+01:00 (duration: 1d 21h 8m 3s)

The command used to launch the workflow was as follows:

  nextflow run nf-core/methylseq -profile docker --reads '/media/holmes/backup-exclude/wg-blimp-
      ↪ eval-blood-sperm-pipes/fastq/*_R{1,2}.fastq.gz' --fasta /media/holmes/genomes/
      ↪ Homo_sapiens.GRCh38.p7/bwameth/assembled.fa --bwa_meth_index /media/holmes/genomes/
      ↪ Homo_sapiens.GRCh38.p7/bwameth/assembled.fa --fasta_index /media/holmes/genomes/
      ↪ Homo_sapiens.GRCh38.p7/bwameth/assembled.fa.fai --max_cpus 64 --aligner bwameth --
      ↪ max_time 2400.h



Pipeline Configuration:
-----------------------
 - Pipeline Release: master
 - Pipeline Name: nf-core/methylseq
 - Run Name: berserk_celsius
 - Reads: /media/holmes/backup-exclude/wg-blimp-eval-blood-sperm-pipes/fastq/*_R{1,2}.fastq.gz
 - Aligner: bwameth
 - Spliced alignment: No
 - SLAM-seq: No
 - Local alignment: No
 - Data Type: Paired-End
 - Genome: false
 - BWA-Meth Index: /media/holmes/genomes/Homo_sapiens.GRCh38.p7/bwameth/assembled.fa*
 - Fasta Ref: /media/holmes/genomes/Homo_sapiens.GRCh38.p7/bwameth/assembled.fa
 - Fasta Index: /media/holmes/genomes/Homo_sapiens.GRCh38.p7/bwameth/assembled.fa.fai
 - Trim R1: 0
 - Trim R2: 0
 - Trim 3' R1: 0
 - Trim 3' R2: 0
 - Deduplication: Yes
 - Directional Mode: Yes
 - All C Contexts: No
 - Save Reference: No
 - Save Trimmed: No
 - Save Unmapped: No
 - Save Intermediates: No
 - Current home: /home/marw
 - Current path: /media/holmes/backup-exclude/wg-blimp-eval-blood-sperm-pipes/nextflow_bwameth
 - Max Resources: 128 GB memory, 64 cpus, 2400.h time per job
 - Container: docker - nfcore/methylseq:1.4
 - Output dir: ./results
```

```
- Launch dir: /media/holmes/backup-exclude/wg-blimp-eval-blood-sperm-pipes/nextflow_bwameth
- Working dir: /media/holmes/backup-exclude/wg-blimp-eval-blood-sperm-pipes/nextflow_bwameth/
    ↪ work
- Script dir: /home/marw/.nextflow/assets/nf-core/methylseq
- User: marw
- Config Profile: docker
- Date Started: 2020-01-20T10:08:25.966636+01:00
- Date Completed: 2020-01-22T07:16:29.439988+01:00
- Pipeline script file path: /home/marw/.nextflow/assets/nf-core/methylseq/main.nf
- Pipeline script hash ID: f6350c2414e08a0f04d816270b4d22dc
- Pipeline repository Git URL: https://github.com/nf-core/methylseq.git
- Pipeline repository Git Commit: 40760ecffbf1d3ce8659712f692454ed88e59324
- Pipeline Git branch/tag: master
- Nextflow Version: 19.10.0
- Nextflow Build: 5170
- Nextflow Compile Timestamp: 21-10-2019 15:07 UTC

--
nf-core/methylseq
https://github.com/nf-core/methylseq
```

We note that this workflow in its current version fails to produce methylation calls for all samples. However, this is a known issue that the developers are already working on, see https://github.com/nf-core/methylseq/issues/139 .

3.7 PiGx configuration

For the analysis using PiGx we used the Docker container bimsbbioinfo/pigx:publication (digest 987e466930d3) and the following configuration:

```
locations:
  input-dir: /media/holmes/backup-exclude/wg-blimp-eval-blood-sperm-pipes/fastq
  output-dir: results/
  genome-dir: /media/holmes/genomes/Homo_sapiens.GRCh38.p7/pigx/

general:
  assembly: hg38
  methylation-calling:
    minimum-coverage: 0
    minimum-quality: 10
  differential-methylation:
    cores: 8
    treatment-groups:
      - ['0', '1']
    annotation:
      cpgIsland_bedfile: genome/cpgIslandExt.hg38.bed.gz
      refGenes_bedfile:  genome/refGene.hg38.bed.gz
      webfetch:   no
tools:
  bismark:
    cores: 16
execution:
  submit-to-cluster: no
  jobs: 16
  nice: 19
  cluster:
    memory: 8G
    stack: 128M
    queue: all
    contact-email: none
```

The sample sheet used by PiGx looks as follows:

```
Read1,Read2,SampleID,Protocol,Treatment
60_Hmp01_blood_young_R1.fastq.gz,60_Hmp01_blood_young_R2.fastq.gz,60_Hmp01_blood_young,WGBS,0
60_Hmp02_blood_old_R1.fastq.gz,60_Hmp02_blood_old_R2.fastq.gz,60_Hmp02_blood_old,WGBS,0
60_Hmp01_sperm_young_R1.fastq.gz,60_Hmp01_sperm_young_R2.fastq.gz,60_Hmp01_sperm_young,WGBS,1
60_Hmp02_sperm_old_R1.fastq.gz,60_Hmp02_sperm_old_R2.fastq.gz,60_Hmp02_sperm_old,WGBS,1
```

We note here that PiGx differential methylation analysis took ∼22 hours to complete. We suspect the multithreading to not work as intended: When assigning 64 cores to differential methylation analysis excessive amounts of RAM were used, resulting in OS process termination. However, even with 8 cores each fork used only ∼13% of a single core's resources, meaning the multithreaded version of this analysis does not effectively utilize multiple cores.

3.8 snakePipes configuration

For snakePipes we set up a conda environment containing the following packages:

```
# packages in environment at /media/holmes/scripts/conda/envs/snakePipes:
#
# Name                      Version              Build    Channel
_libgcc_mutex               0.1                   main      conda-forge
aioeasywebdav               2.4.0            py36_1000      conda-forge
aiohttp                     3.6.2       py36h516909a_0      conda-forge
appdirs                     1.4.3                 py_1      conda-forge
async-timeout               3.0.1              py_1000      conda-forge
attrs                       19.3.0                py_0      conda-forge
bcrypt                      3.1.7       py36h516909a_0      conda-forge
boto3                       1.10.46               py_0      conda-forge
botocore                    1.13.46               py_0      conda-forge
bzip2                       1.0.8           h516909a_2      conda-forge
ca-certificates             2019.11.28      hecc5488_0      conda-forge
cachetools                  3.1.1                 py_0      conda-forge
cairo                       1.16.0       hfb77d84_1002      conda-forge
certifi                     2019.11.28          py36_0      conda-forge
cffi                        1.13.2      py36h8022711_0      conda-forge
chardet                     3.0.4            py36_1003      conda-forge
configargparse              0.13.0                py_1      conda-forge
cryptography                2.8         py36h72c5cf5_1      conda-forge
cython                      0.29.14     py36he1b5a44_0      conda-forge
datrie                      0.8         py36h516909a_0      conda-forge
decorator                   4.4.1                 py_0      conda-forge
docutils                    0.15.2              py36_0      conda-forge
dropbox                     9.4.0                 py_0      conda-forge
expat                       2.2.5        he1b5a44_1004      conda-forge
fftw                        3.3.8       nompi_h7f3a6c3_1110    conda-forge
filechunkio                 1.6                 py36_0      bioconda
font-ttf-dejavu-sans-mono   2.37            hab24e00_0      conda-forge
font-ttf-inconsolata        2.001           hab24e00_0      conda-forge
font-ttf-source-code-pro    2.030           hab24e00_0      conda-forge
font-ttf-ubuntu             0.83            hab24e00_0      conda-forge
fontconfig                  2.13.1       h86ecdb6_1001      conda-forge
fonts-conda-forge           1                        0      conda-forge
freetype                    2.10.0          he983fc9_1      conda-forge
fribidi                     1.0.5        h516909a_1002      conda-forge
ftputil                     3.2                 py36_0      bioconda
fuzzywuzzy                  0.17.0                py_0      conda-forge
gdk-pixbuf                  2.32.2                   1      bioconda
gettext                     0.19.8.1     hc5be6a0_1002      conda-forge
ghostscript                 9.18                     1      bioconda
giflib                      5.2.1           h516909a_1      conda-forge
gitdb2                      2.0.6                 py_0      conda-forge
gitpython                   3.0.5                 py_0      conda-forge
glib                        2.58.3      py36h6f030ca_1002    conda-forge
gobject-introspection       1.58.2      py36h5503ade_1002    conda-forge
google-api-core             1.15.0              py36_0      conda-forge
google-auth                 1.10.0                py_0      conda-forge
google-cloud-core           1.1.0                 py_0      conda-forge
google-cloud-storage        1.20.0                py_0      conda-forge
google-resumable-media      0.4.1                 py_0      conda-forge
googleapis-common-protos    1.6.0               py36_0      conda-forge
graphite2                   1.3.13       hf484d3e_1000      conda-forge
graphviz                    2.42.3          h0511662_0      conda-forge
harfbuzz                    2.4.0           h9f30f68_3      conda-forge
icu                         64.2            he1b5a44_1      conda-forge
idna                        2.8              py36_1000      conda-forge
idna_ssl                    1.1.0            py36_1000      conda-forge
imagemagick                 7.0.9_14      pl526ha9fe49d_1   conda-forge
importlib_metadata          1.3.0               py36_0      conda-forge
jbig                        2.1          h14c3975_2001      conda-forge
jinja2                      2.10.3                py_0      conda-forge
jmespath                    0.9.4                 py_0      conda-forge
jpeg                        9c           h14c3975_1001      conda-forge
jsonschema                  3.2.0               py36_0      conda-forge
libblas                     3.8.0          14_openblas      conda-forge
libcblas                    3.8.0          14_openblas      conda-forge
libcroco                    0.6.13          h8d621e5_0      conda-forge
libffi                      3.2.1        he1b5a44_1006      conda-forge
libgcc                      7.2.0           h69d50b8_2      conda-forge
libgcc-ng                   9.2.0           hdf63c60_0      conda-forge
libgfortran-ng              7.3.0           hdf63c60_2      conda-forge
libiconv                    1.15         h516909a_1005      conda-forge
liblapack                   3.8.0          14_openblas      conda-forge
libopenblas                 0.3.7           h5ec1e0e_6      conda-forge
libpng                      1.6.37          hed695b0_0      conda-forge
libprotobuf                 3.11.2          h8b12597_0      conda-forge
librsvg                     2.46.4          h33a7fed_0      conda-forge
libstdcxx-ng                9.2.0           hdf63c60_0      conda-forge
libtiff                     4.1.0           hc3755c2_3      conda-forge
```

```
libtool                 2.4.6              h14c3975_1002    conda-forge
libuuid                 2.32.1             h14c3975_1000    conda-forge
libwebp                 1.0.2                 h56121f0_5    conda-forge
libxcb                  1.13               h14c3975_1002    conda-forge
libxml2                 2.9.10                hee79883_0    conda-forge
lz4-c                   1.8.3              he1b5a44_1001    conda-forge
markupsafe              1.1.1            py36h516909a_0    conda-forge
more-itertools          8.0.2                       py_0    conda-forge
multidict               4.7.3            py36h516909a_0    conda-forge
ncurses                 6.1                hf484d3e_1002    conda-forge
networkx                2.4                         py_0    conda-forge
nose                    1.3.7                 py36_1003    conda-forge
numpy                   1.17.3           py36h95a1406_0    conda-forge
openjpeg                2.3.1                 h981e76c_3    conda-forge
openssl                 1.1.1d                h516909a_0    conda-forge
pandas                  0.25.3           py36hb3f55d8_0    conda-forge
pango                   1.42.4                ha030887_1    conda-forge
paramiko                2.7.1                    py36_0    conda-forge
pcre                    8.43                  he1b5a44_0    conda-forge
perl                    5.26.2             h516909a_1006    conda-forge
pip                     19.3.1                   py36_0    conda-forge
pixman                  0.38.0             h516909a_1003    conda-forge
pkg-config              0.29.2             h516909a_1006    conda-forge
prettytable             0.7.2                       py_3    conda-forge
protobuf                3.11.2           py36he1b5a44_0    conda-forge
psutil                  5.6.7            py36h516909a_0    conda-forge
pthread-stubs           0.4                h14c3975_1001    conda-forge
pyasn1                  0.4.8                       py_0    conda-forge
pyasn1-modules          0.2.7                       py_0    conda-forge
pycparser               2.19                     py36_1    conda-forge
pygments                2.5.2                       py_0    conda-forge
pygraphviz              1.3.1                    py36_0    bioconda
pynacl                  1.3.0          py36h516909a_1001    conda-forge
pyopenssl               19.1.0                   py36_0    conda-forge
pyrsistent              0.15.6           py36h516909a_0    conda-forge
pysftp                  0.2.9                    py36_0    bioconda
pysocks                 1.7.1                    py36_0    conda-forge
python                  3.6.7              h357f687_1006    conda-forge
python-dateutil         2.8.1                       py_0    conda-forge
python-irodsclient      0.7.0                       py_0    conda-forge
python-levenshtein      0.12.0                   pypi_0    pypi
pytz                    2019.3                      py_0    conda-forge
pyyaml                  5.2              py36h516909a_0    conda-forge
ratelimiter             1.2.0                 py36_1000    conda-forge
readline                8.0                   hf8c457e_0    conda-forge
requests                2.22.0                   py36_1    conda-forge
rsa                     3.1.4                    py36_0    bioconda
s3transfer              0.2.1                    py36_0    conda-forge
setuptools              44.0.0                   py36_0    conda-forge
six                     1.13.0                   py36_0    conda-forge
smmap2                  2.0.5                       py_0    conda-forge
snakemake               5.9.1                          0    bioconda
snakemake-minimal       5.9.1                       py_0    bioconda
snakepipes              1.3.1                       py_0    mpi-ie
sqlite                  3.30.1                hcee41ef_0    conda-forge
tk                      8.6.10                hed695b0_0    conda-forge
typing_extensions       3.7.4.1                  py36_0    conda-forge
urllib3                 1.25.7                   py36_0    conda-forge
wheel                   0.33.6                   py36_0    conda-forge
wrapt                   1.11.2           py36h516909a_0    conda-forge
xmlrunner               1.7.7                       py_0    conda-forge
xorg-kbproto            1.0.7              h14c3975_1002    conda-forge
xorg-libice             1.0.10                h516909a_0    conda-forge
xorg-libsm              1.2.3              h84519dc_1000    conda-forge
xorg-libx11             1.6.9                 h516909a_0    conda-forge
xorg-libxau             1.0.9                 h14c3975_0    conda-forge
xorg-libxdmcp           1.1.3                 h516909a_0    conda-forge
xorg-libxext            1.3.4                 h516909a_0    conda-forge
xorg-libxpm             3.5.13                h516909a_0    conda-forge
xorg-libxrender         0.9.10             h516909a_1002    conda-forge
xorg-libxt              1.1.5              h516909a_1003    conda-forge
xorg-renderproto        0.11.1             h14c3975_1002    conda-forge
xorg-xextproto          7.3.0              h14c3975_1002    conda-forge
xorg-xproto             7.0.31             h14c3975_1007    conda-forge
xz                      5.2.4              h14c3975_1001    conda-forge
yaml                    0.2.2                 h516909a_1    conda-forge
yarl                    1.3.0          py36h516909a_1000    conda-forge
zipp                    0.6.0                       py_0    conda-forge
zlib                    1.2.11             h516909a_1006    conda-forge
zstd                    1.4.4                 h3b9ef0a_1    conda-forge
```

The following command was used to invoke the snakePipes workflow:

```
WGBS -c config.yaml --local -j 64 --sampleSheet samples.txt --reads _R1 _R2 -i /media/holmes/
    ↪ backup-exclude/wg-blimp-eval-blood-sperm-pipes/fastq -o . config.yaml
```

The configuration file looks as follows:

```
## General/Snakemake parameters, only used/set by wrapper or in Snakemake cmdl, but not in
    ↪ Snakefile
genome_fasta: /media/holmes/genomes/Homo_sapiens.GRCh38.p7/assembled.fa
genome_index: /media/holmes/genomes/Homo_sapiens.GRCh38.p7/assembled.fa.fai
genome_2bit: /media/holmes/genomes/Homo_sapiens.GRCh38.p7/assembled.fa
genes_gtf: /media/holmes/genomes/Homo_sapiens.GRCh38.98/Homo_sapiens.GRCh38.98.gtf
bwameth_index: /media/holmes/genomes/Homo_sapiens.GRCh38.p7/assembled.fa
genome_size: 2900338458
tempDir: /tmp/
snakemakeOptions: --use-conda --conda-prefix conda
```

The sample file was set to:

```
name      condition
60_Hmp01_blood_young      blood
60_Hmp01_sperm_young      sperm
60_Hmp02_blood_old        blood
60_Hmp02_sperm_old        sperm
```

We note that snakePipes did not successfully perform DMR calling using our configuration. While no explicit error was thrown, the DMR calling process seemed to have frozen, as `strace` did not report any activity. We unsuccessfully waited for >450 hours for the process to complete and speculate that some conda dependencies were pulling wrong system libraries, resulting in processes hanging. As this is an issue that might also affect wg-blimp in the future, we provide a Docker container as a backup in case of issues with the Bioconda installation.

References

[1] Wallner S, Schröder C, Leitão E, Berulava T, Haak C, Beißer D, et al. Epigenetic dynamics of monocyte-to-macrophage differentiation. Epigenetics & Chromatin. 2016 Jul;9(1):33. Available from: https://doi.org/10.1186/s13072-016-0079-z.

[2] Laurentino S, Cremers JF, Horsthemke B, Tuettelmann F, Czeloth K, Zitzmann M, et al. Healthy ageing men have normal reproductive function but display germline-specific molecular changes. medRxiv. 2019;Available from: https://www.medrxiv.org/content/early/2019/09/16/19006221.

[3] Schlaeger TM, Daheron L, Brickler TR, Entwisle S, Chan K, Cianci A, et al. A comparison of non-integrating reprogramming methods. Nature biotechnology. 2015;33(1):58.

[4] Burger L, Gaidatzis D, Schübeler D, Stadler MB. Identification of active regulatory regions from DNA methylation data. Nucleic Acids Research. 2013 07;41(16):e155–e155. Available from: https://doi.org/10.1093/nar/gkt599.
